# Diagnosis and Treatment of Neurocysticercosis: 2017 Clinical Practice Guidelines by the Infectious Diseases Society of America (IDSA) and the American Society of Tropical Medicine and Hygiene (ASTMH)

**DOI:** 10.4269/ajtmh.18-88751

**Published:** 2018-04

**Authors:** A. Clinton White, Christina M. Coyle, Vedantam Rajshekhar, Gagandeep Singh, W. Allen Hauser, Aaron Mohanty, Hector H. Garcia, Theodore E. Nash

**Affiliations:** 1 University of Texas Medical Branch, Galveston, Texas;; 2 Albert Einstein College of Medicine, Bronx, New York;; 3 Christian Medical College, Vellore, India;; 4 Dayanand Medical College, Ludhiana, India;; 5 Columbia University, New York, New York;; 6 University of Texas Medical Branch, Galveston, Texas;; 7 Instituto Nacional de Ciencias Neurologicas, Lima, Peru;; 8 Universidad Peruana Cayetano Heredia, Lima, Peru;; 9 National Institutes of Health, Bethesda, Maryland

## EXECUTIVE SUMMARY

Guidelines for the clinical management of patients with neurocysticercosis (NCC) were prepared by a panel of the Infectious Diseases Society of America (IDSA) and the American Society of Tropical Medicine and Hygiene (ASTMH). The guidelines are intended for infectious disease specialists, neurologists, neurological surgeons, internists, pediatricians, and family practitioners.

**Figure 1.**
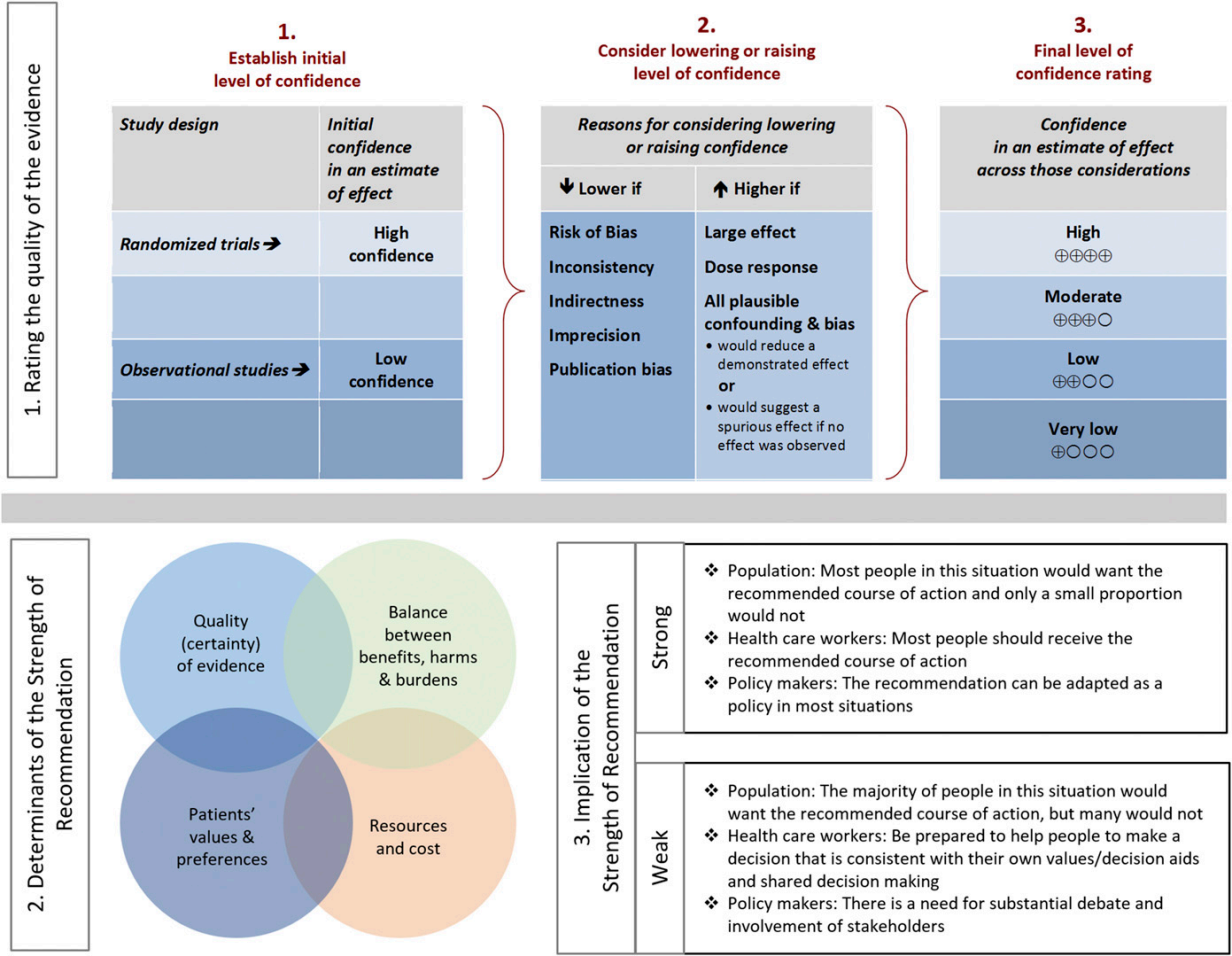
Approach and implications to rating the quality of evidence and strength of recommendations using the Grading of Recommendations, Assessment, Development and Evaluation (GRADE) methodology (unrestricted use of the figure granted by the U.S. GRADE Network).

These guidelines present our approaches to the diagnosis and management of patients with the different forms of NCC, including viable parenchymal NCC (VPN), single enhancing lesions (SEL), calcified parenchymal NCC (CPN), ventricular NCC (IVN), and subarachnoid NCC (SAN). Our recommendations are based on the best evidence available. Because of the complex variations in clinical manifestations and the limitations of the literature, many of the recommendations are based on observational studies, anecdotal data, or expert opinion rather than randomized clinical trials. The approaches we describe are intended to be both applicable and feasible in the United States and Canada (for simplicity, referred to here as North America). The recommendations may not apply for settings where resource constraints may limit their applicability. The executive summary in the following paragraphs lists the recommendations for the diagnosis and clinical management of NCC. A detailed description of the methods, background, and evidence summaries that support each of the recommendations can be found online in the full text of the guidelines. A criterion for grading evidence is presented in Figure 1.^1^ Note that diagnosis and management of patients with NCC can be challenging even with expert guidelines. Because of this complexity, clinicians with little experience with this disease should have a low threshold for consultation with an expert in the disease.

## RECOMMENDATIONS FOR DIAGNOSIS AND BASELINE EVALUATION

### I. How should NCC be diagnosed?

#### Recommendations.

1. Although there is a wide range of clinical manifestations of NCC, the two most common clinical presentations are with seizures and increased intracranial pressure (fact, no grade).
2. Initial evaluation should include careful history and physical examination, and neuroimaging studies (fact, no grade).
3. We recommend serologic testing with enzyme-linked immunotransfer blot (EITB) as a confirmatory test in patients with suspected NCC (strong, moderate). Enzyme-linked immunosorbent assay (ELISA) tests using crude antigens should be avoided because of poor sensitivity and specificity (strong, moderate).

### II. What imaging studies should be used to classify disease?

#### Recommendation.

4. We recommend both a brain magnetic resonance imaging (MRI) and a non-contrast computed tomography (CT) scan for classifying patients with newly diagnosed NCC (strong, moderate).

### III. What additional tests should be performed before initiation of therapy?

#### Recommendations.

5. We suggest screening for latent tuberculosis infection in patients likely to require prolonged corticosteroids (weak, low).
6. We suggest screening or empiric therapy for *Strongyloides stercoralis* in patients likely to require prolonged corticosteroids (weak, low).
7. We recommend that all patients with NCC undergo a fundoscopic examination before initiation of anthelminthic therapy (strong, moderate).
8. We suggest that patients with NCC who has probably acquired NCC in a non-endemic area have their household members be screened for tapeworm carriage (weak, low). *Remark*: This is a public health issue and can often be addressed by the local health department.

### IV. How should antiparasitic and anti-inflammatory therapy be monitored?

#### Recommendations.

9. We recommend that patients treated with albendazole for more than 14 days be monitored for hepatotoxicity and leucopenia (strong, moderate).
10. No additional monitoring is needed for patients receiving combination therapy with albendazole and praziquantel beyond that recommended for albendazole monotherapy (strong, moderate).

## RECOMMENDATIONS FOR THE TREATMENT OF VIABLE INTRAPARENCHYMAL NCC (VPN)

### V. What is the role of antiparasitic drugs in VPN?

#### Recommendations.

11. In patients with untreated hydrocephalus or diffuse cerebral edema, we recommend management of elevated intracranial pressure alone and not antiparasitic treatment (strong, moderate). *Remarks*: The management of patients with diffuse cerebral edema should be anti-inflammatory therapy such as corticosteroids, whereas hydrocephalus usually requires a surgical approach.
12. In the absence of elevated intracranial pressure, we recommend the use of antiparasitic drugs in all patients with VPN (strong, moderate).
13. For patients with one to two viable parenchymal cysticerci, we recommend albendazole monotherapy for 10–14 days compared with either no antiparasitic therapy (strong, high) or combination antiparasitic therapy (weak, moderate). *Remarks*: The usual dose of albendazole is 15 mg/kg/day divided into two daily doses for 10–14 days with food. We recommend a maximum dose of 1,200 mg/day.
14. We recommend albendazole (15 mg/kg/day) combined with praziquantel (50 mg/kg/day) for 10–14 days rather than albendazole monotherapy for patients with more than two viable parenchymal cysticerci (strong, moderate).
15. We suggest retreatment with antiparasitic therapy for parenchymal cystic lesions persisting for 6 months after the end of the initial course of therapy (weak, low).

### VI. What is the role of anti-inflammatory therapy in management of VPN?

#### Recommendation.

16. We recommend adjunctive corticosteroid therapy begun before antiparasitic drugs rather than no adjunctive therapy in all patients treated with antiparasitic therapy (strong, moderate).

### VII. What is the role of antiepileptic drugs in VPN?

#### Recommendations.

17. We recommend antiepileptic drugs in all NCC patients with seizures (strong, low).
18. In patients with few seizures before antiparasitic therapy, resolution of the cystic lesion on imaging studies, and no seizures for 24 consecutive months, we suggest that tapering off and stopping antiepileptic drugs be considered (weak, moderate).
19. In the absence of controlled data, the choice of antiepileptic drugs should be guided by local availability, cost, drug interactions, and potential side effects (fact, no grade).

### VIII. What follow-up is recommended after initial antiparasitic therapy for patients with VPN?

#### Recommendation.

20. We suggest that MRI be repeated at least every 6 months until resolution of the cystic component (strong, low).

## RECOMMENDATIONS FOR THE TREATMENT OF DEGENERATING INTRAPARENCHYMAL NCC INCLUDING PATIENTS WITH SEL DUE TO NCC (SEL, ALSO TERMED SOLITARY CYSTICERCUS GRANULOMA)

### IX. What should be the initial approach to the patient with multiple enhancing lesions from NCC?

#### Recommendation.

21. We recommend that patients with multiple enhancing lesions and seizures be initially treated with antiepileptic drugs, antiparasitic therapy, and corticosteroids as outlined in the section on viable parenchymal cysticerci (weak, moderate).

### X. What is the role of antiepileptic medications in patients with SEL from cysticercosis with seizures?

#### Recommendations.

22. We recommend antiepileptic drugs for all patients with SEL and seizures (strong, moderate).
23. In the absence of controlled data, the choice of antiepileptic drugs can be guided by local availability, cost, drug interactions, and potential side effects (fact, no grade).
24. In patients who have been seizure free for 6 months, we suggest tapering off and stopping antiepileptic drugs after resolution of the lesion in patients with SEL without risk factors for recurrent seizures (weak, moderate). *Remark*: Risk factors for recurrent seizures include residual cystic lesions or calcifications on neuroimaging studies, breakthrough seizures, or more than two seizures.

### XI. What is the role of antiparasitic drugs in patients with SEL?

#### Recommendation.

25. We suggest albendazole therapy rather than no antiparasitic therapy for all patients with SEL (weak, moderate). *Remarks*: Albendazole (15 mg/kg/day in twice daily doses up for 1–2 weeks) with meals.

### XII. What is the role of anti-inflammatory therapy in SEL?

#### Recommendation.

26. We recommend that patients with SEL treated with antiparasitic drugs should also be treated with corticosteroids initiated before antiparasitic therapy (strong, moderate).

### XIII. How should patients with SEL be followed?

#### Recommendation.

27. We suggest that MRI be repeated at least every 6 months until resolution of cystic lesions for patients with SEL (weak, low).

## RECOMMENDATIONS FOR THE TREATMENT OF CALCIFIED PARENCHYMAL NEUROCYSTICERCOSIS (CPN)

### XIV. What should the initial approach be to patients with calcified lesions suggestive of CPN?

#### Recommendation.

28. We suggest brain MRI in patients with seizures or hydrocephalus and only CPN on CT (weak, low).

### XV. What is the role of antiparasitic drugs, antiepileptic drugs, and anti-inflammatory medications in the management of patients with CPN?

#### Recommendations.

29. We recommend symptomatic therapy alone instead of antiparasitic drugs in patients with calcified parenchymal lesions (strong, moderate).
30. We suggest that corticosteroids not be routinely used in patients with isolated CPN and perilesional edema (weak, low).

### XVI. Is there a role for surgical therapy in refractory cases?

#### Recommendation.

31. In patients with refractory epilepsy and CPN, we suggest evaluation for surgical removal of seizure foci (weak, low).

## RECOMMENDATIONS FOR THE TREATMENT OF INTRAVENTRICULAR NEUROCYSTICERCOSIS (IVN)

### XVII. How are extra-parenchymal cysts best identified?

#### Recommendation.

32. We recommend MRI with three-dimensional (3D) volumetric sequencing to identify intraventricular and subarachnoid cysticerci in patients with hydrocephalus and suspected NCC (strong, moderate).

### XVIII. What is the optimal approach to management of IVN in the lateral and third ventricles?

#### Recommendation.

33. When possible, we recommend removal of the cysticerci by minimally invasive, neuroendoscopy over other surgical or medical approaches for cysticerci of the lateral and third ventricles (Strong, moderate). *Remark*: Most experts recommend that antiparasitic drugs not be used preoperatively because such treatment could result in disruption of parasite integrity and an inflammatory response that could prevent successful cyst removal.

### XIX. What is the optimal surgical approach to management of IVN in the fourth ventricle?

#### Recommendation.

34. In cases in which surgical removal of fourth ventricular cysticerci is possible, we recommend surgical removal rather than medical therapy and/or shunt surgery (strong, moderate).

### XX. What is the optimal approach to adherent IVN?

#### Recommendation.

35. We suggest shunt surgery for hydrocephalus rather than cyst removal when surgical removal is technically difficult (weak, low). *Remark*: Attempted removal of inflamed or adherence ventricular cysticerci is associated with increased risk of complications.

### XXI. Does medical therapy as an adjunct to procedures or as primary therapy have an impact on outcome in treating patients with IVN?

#### Recommendations.

36. We recommend corticosteroids to decrease brain edema in the perioperative period (fact, no grade).
37. We suggest antiparasitic drugs with corticosteroid therapy after shunt insertion to decrease subsequent shunt failure in patients in whom surgical removal of isolated intraventricular cysts is not possible (weak, low) but neither after successful removal of intraventricular cysts (weak, low). *Remark*: Note that intraventricular cysts may be accompanied by other lesions with indications for antiparasitic therapy.

## RECOMMENDATIONS FOR SUBARACHNOID NEUROCYSTICERCOSIS (SAN)

### XXII. What is the role of medical therapy in SAN in the basilar cisterns or Sylvian fissures?

#### Recommendations.

38. We recommend that patients with subarachnoid cysts be treated with antiparasitic drugs (strong, low).
39. We suggest that antiparasitic therapy be continued until there is radiologic resolution of viable cysticerci on MRI and resolution of other evidence of cysticerci (weak, low). Responses often require prolonged therapy, which can last for over a year.
40. We recommend anti-inflammatory therapy (such as high-dose corticosteroids) for SAN initiated before antiparasitic drugs (strong, moderate).
41. We suggest that methotrexate be considered as a steroid-sparing agent in patients requiring prolonged courses of anti-inflammatory therapy (weak, low).

### XXIII. What is the role of neurosurgery in SAN?

#### Recommendation.

42. We recommend that patients with hydrocephalus from SAN be treated with shunt surgery in addition to medical therapy (strong, low).
43. We suggest that some patients may benefit from surgical debulking over shunt surgery alone (weak, low).

## RECOMMENDATIONS FOR SPINAL NCC (SN)

### XXIV. How is SN best treated?

#### Recommendations.

44. We recommend corticosteroid treatment of patients with SN with evidence of spinal cord dysfunction (e.g., paraparesis or incontinence) or as adjunctive therapy along with antiparasitic therapy (strong, moderate).
45. We suggest that both medical (antiparasitic drugs plus anti-inflammatory drugs) and surgical approaches be considered for SN (weak, low). *Practice Statement*: There are anecdotes of good responses of SN to medical and/or surgical therapy. However, there are no good data supporting one approach over the other. We suggest that management of SN should be individualized based on symptoms, location of the cysticerci, degree of arachnoiditis, and surgical experience. Recommendations for antiparasitic drugs, reimaging, and follow-up of SAN should also be considered for subarachnoid SN.

## RECOMMENDATIONS FOR MANAGEMENT OF OCULAR CYSTICERCOSIS (OC)

### XXV. What is the optimal management of OC?

#### Recommendation.

46. We suggest that intraocular cysticerci should be treated with surgical removal rather than with antiparasitic drugs (weak, low).

## RECOMMENDATIONS FOR THE TREATMENT OF SPECIAL POPULATIONS (SP)

### XXVI. Should children be managed differently from adults?

#### Recommendation.

47. There is no evidence that management of NCC in children should be different from adults with the same form of disease (strong, moderate). Dosing should be weight based.

### XXVII. Should management be different in pregnant women?

#### Recommendation.

48. We suggest that antihelminthic therapy should be deferred until after pregnancy (weak, low). *Remarks*: Pregnant patients with elevated intracranial pressure need to be aggressively managed as they would be if not pregnant. Corticosteroids can be used in pregnancy when necessary. The use of antiepileptic drugs should take into account altered pharmacokinetics and potential teratogenicity. Phenobarbital and valproic acid are known to have high rates of teratogenicity. Antihelminthic drugs are rarely required emergently and their use can usually be deferred until after delivery. Methotrexate is teratogenic and should be avoided.

## INTRODUCTION

In the first section, the panel summarizes background information relevant to the topic. In the second section, the panel poses questions regarding the diagnosis and treatment of NCC, evaluates applicable clinical trial and observational data, and makes recommendations using the Grading of Recommendations, Assessment, Development and Evaluation (GRADE) framework.^1^ The following 27 clinical questions were answered:

I. How should NCC be diagnosed?
II. What imaging studies should be used to classify disease?
III. What additional tests should be performed before initiation of therapy?
IV. How should antiparasitic and anti-inflammatory therapy be monitored?
V. What is the role of antiparasitic drugs in VPN?
VI. What is the role of anti-inflammatory therapy in management of VPN?
VII. What is the role of antiepileptic drugs in VPN?
VIII. What follow-up is recommended after initial antiparasitic therapy for patients with VPN?
IX. What should be the initial approach to the patient with multiple enhancing lesions from NCC?
X. What is the role of antiepileptic medications in patients with SEL from cysticercosis with seizures?
XI. What is the role of antiparasitic drugs in patients with SEL?
XII. What is the role of anti-inflammatory therapy in SEL?
XIII. How should patients with SEL be followed?
XIV. What should the initial approach be to patients with calcified lesions suggestive of CPN?
XV. What is the role of antiparasitic drugs, antiepileptic drugs, and anti-inflammatory medications in the management of patients with CPN?
XVI. Is there a role for surgical therapy in refractory cases?
XVII. How are extra parenchymal cysts best identified?
XVIII. What is the optimal approach to management of IVN in the lateral and third ventricles?
XIX. What is the optimal surgical approach to management of IVN in the fourth ventricles?
XX. What is the optimal approach to adherent IVN?
XXI. Does medical therapy as an adjunct to procedures or as primary therapy have an impact on outcome in treating patients with IVN?
XXII. What is the role of medical therapy in SAN in the basilar cisterns or Sylvian fissures?
XXIII. What is the role of neurosurgery in SAN?
XXIV. How is SN best treated?
XXV. What is the optimal management of OC?
XXVI. Should children be managed differently from adults?
XXVII. Should management be different in pregnant women?

## BACKGROUND

NCC, caused by the larval form of the cestode parasite *Taenia solium*, is a major cause of seizure and neurologic disease worldwide and is common among immigrant populations in the United States. Highly endemic regions include Latin America, sub-Saharan Africa, and parts of Asia.^2,3^ In endemic areas, it is linked to approximately 29% of cases of seizures.^4^ Estimates suggest that there are more than 2,000 cases per year in the United States with hospital charges of nearly $100 million per year.^5,6^ Humans can be hosts to both the tapeworm form and larval forms of the parasite. Taeniasis (also termed taeniosis) refers to infestation of the human intestines with the tapeworm form. The tapeworm is acquired by ingestion of undercooked pork. The scolex evaginates and attaches to the intestinal wall and segments termed proglottids form a long ribbon-like chain referred to as the strobili. The gravid proglottids and eggs are passed in stool. Humans can also develop cysticercosis after ingestion of ova. Cysticercosis refers to infection of the tissues with the larval cyst (or metacestode). Normally, pigs host the cysts, which are acquired by ingestion of ova or proglottids from human feces. However, humans can be infected by ingestion of ova and develop cysticercosis. Neurocysticercosis refers to cysticercosis involving the central nervous system, including the brain parenchyma, ventricles, basilar cisterns, sulci, gyri, spine, and retina.

The pathogenesis, natural history, clinical manifestations, and management vary with the location of the cysticerci.^3^ For example, the main clinical manifestations vary between different forms of disease (Table 1). Parenchymal NCC typically presents with seizures or headache. Ventricular neurocysticercosis most often presents with obstructive hydrocephalus. Subarachnoid neurocysticercosis can present with communicating hydrocephalus, meningitis, stroke, or focal neurologic findings. Mixed forms are also common. Because of the complexity of diagnosis and management, the American Society of Tropical Medicine and Hygiene (ASTMH) and the Infectious Diseases Society of America (IDSA) agreed to jointly develop guidelines for the diagnosis and management of NCC.

## METHODOLOGY

### Panel composition.

The IDSA and the ASTMH convened experts in the diagnosis and management of NCC from the fields of tropical and infectious diseases, neurology, and neurosurgery, including experts coming from endemic areas as well as from North America. All panel members were selected on the basis of their clinical expertise in NCC and their expertise in the disciplines of infectious diseases, tropical medicine, neurology, and neurosurgery.

### Evidence review—the GRADE method.

GRADE is a systematic approach to guideline development that has been described in detail elsewhere.^1,7^ The IDSA adopted GRADE in 2008. In the GRADE system, the guideline panel assigns each recommendation with separate ratings for the underlying quality of evidence supporting the recommendation and for the strength with which the recommendation is made (Figure 1). Data from randomized controlled trials begin as “high” quality, and data from observational studies begin as “low” quality. However, the panel may judge that specific features of the data warrant decreasing or increasing the quality of evidence rating, and GRADE provides guidance on how such factors should be weighed.^7^ The strength assigned to a recommendation chiefly reflects the panel’s confidence that the benefits of following the recommendation are likely to outweigh potential harms. Although the quality of evidence is an important factor in choosing recommendation strength, it is not prescriptive.

### Process overview.

Panel members were each assigned to review the recent literature for at least one topic, evaluate the evidence, determine the strength of recommendations, and develop written evidence in support of these recommendations. The panel had several in-person meetings and conducted most of its work through monthly teleconferences and electronically based discussion during 2011–2017. Recommendations and grading of evidence were developed by the panel members based on GRADE criteria. All members of the panel participated in the preparation and/or review of the draft guidelines.

### Conflicts of interests.

Members of the expert panel complied with the IDSA policy regarding conflicts of interest, which requires disclosure of any financial or other interest that might be construed as constituting an actual, potential, or apparent conflict. IDSA provided a conflict of interest disclosure statement to panel members and asked them to identify ties to companies manufacturing or developing products that might be affected by promulgation of the guideline. Information was requested regarding employment, consultancies, stock ownership, honoraria, research funding, expert testimony, and membership on company advisory committees. Regular updates of information pertaining to conflicts of interest were requested from each panel member after scheduled teleconference meetings. The panel made decisions on a case-by-case basis as to whether an individual’s role should be limited as a result of a conflict. No limiting conflicts were identified. Complying with IDSA policy, most of the panel members were free of conflicts and one of the chairs was free of all conflicts.

### Review and approval process.

The panel obtained feedback from three external peer reviewers. The final document was reviewed and approved by the entire panel. The contents of the guidelines and manuscript was reviewed and approved by the IDSA Standards and Practice Guidelines Committee and the Board of Directors of the IDSA and ASTMH before dissemination.

### Future guideline revisions.

At annual intervals, the panel chairs will be asked for their input on the need to update the guideline based on an examination of the present literature. The Standards and Practice Guidelines Committee of the IDSA will consider this input and determine the necessity and timing of an update. If warranted, the entire panel or a subset thereof will be convened to discuss potential changes.

## BACKGROUND INFORMATION ON CYSTICERCOSIS

More than 2,000 cases of NCC are diagnosed each year in the United States.^5,6^ Epidemiologic studies suggest that NCC is the cause of approximately 29% of seizures in endemic areas and about 2% of patients presenting with seizures presenting to U.S. emergency rooms.^2–5,8–10^ The seizures can be focal, focal with generalization, or generalized. Thus, NCC should be considered in all patients with seizures potentially exposed to a tapeworm carrier. Increased intracranial pressure is also a common manifestation of NCC. Approximately 20% of cases present with increased intracranial pressure, mainly obstructive hydrocephalus.^2–4,8,10,11^

A wide range of additional neurologic symptoms may be the initial symptoms of NCC. Patients can present with headaches, including migraine headaches. Less common manifestations include spinal radiculopathies, cerebrovascular accidents (lacunar infarctions, thrombotic, and hemorrhagic strokes), visual changes, and mass lesions.

## RECOMMENDATIONS FOR DIAGNOSIS AND BASELINE EVALUATION

### I. How should NCC be diagnosed?

#### Recommendations.

1. Although there is a wide range of clinical manifestations of NCC, the two most common clinical presentations are seizures and increased intracranial pressure (fact, no grade).
2. Initial evaluation should include careful history and physical examinations, and neuroimaging studies (fact, no grade).
3. We recommend serologic testing with EITB as a confirmatory test in patients with suspected NCC (strong, moderate). Enzyme-linked immunosorbent assay tests using crude antigens should be avoided because of poor sensitivity and specificity (strong, moderate).

#### Evidence summary.

All patients with suspected NCC should undergo a thorough history and physical examination with particular attention to exposure. Because there is typically a latent period of years (months to decades) between infection and onset of symptoms, exposure history should not be limited to recent periods. Because there is marked variability in exposure within given countries, this should not be limited to the country of origin or residence but also include queries about access to safe water and improved sanitation throughout life, contact with tapeworm carriers, and contact with pork-raising areas (especially among family and neighbors), which may have occurred months to years before onset of symptoms. Because tapeworm carriers can infect themselves, patients should also be queried about consumption of undercooked pork or passage of tapeworm segments. A thorough history should include query about symptoms of diseases that might be confused with NCC (e.g., fever, night sweats, and weight loss would suggest tuberculosis). Examination should pay careful attention to signs of diseases that could be confused with NCC (e.g., regional adenopathy may suggest tuberculosis or a malignancy).^12^

All patients with suspected NCC should undergo neuroimaging.^13,14^ CT is generally more sensitive at detecting calcified lesions and MRI is more sensitive for detection of the scolex, edema, small parenchymal lesions, posterior fossa lesions, and involvement of the subarachnoid spaces and ventricles. Fluid attenuation inversion recovery (FLAIR) sequences are particularly helpful for identifying associated edema and the scolex.^14,15^

There are a number of causes of cystic lesions on radiographic studies that can have a similar appearance to NCC. These include a number of infections, particularly tuberculomas, brain abscesses, or occasionally parasitic lesions (*Echinococcus granulosus* and *Paragonimus* species). Tumors can also resemble NCC (including metastatic lesions, primary brain cancers or lymphoma, and histiocytosis). Often, the parasite scolex is visible as an intra-cystic nodule, typically round to slightly elongated, 1–2 mm in diameter, and isodense or slightly more dense than brain parenchyma on CT or T1 imaging.

If a scolex is definitely identified, the diagnosis is certain. However, there are a number of artifacts that can be confused with a scolex. Parenchymal cysticerci are round. Most are between 5 and 20 mm in diameter, but they can be larger, especially if located in the gyri and fissures. Parenchymal lesions with cystic areas diameter greater than 20 mm, with irregular borders, or accompanied by midline shift are more likely to have other causes.^12^ Midline shift is usually limited to larger cysts. Symptoms and signs of systemic illnesses (evidence of a primary tumor, fevers, night sweats, weight loss, and adenopathy) also make NCC less likely.

The serologic antibody test of choice is the EITB using parasite glycoproteins (available from Centers for Disease Control and some reference laboratories) performed on serum.^16,17^ Enzyme-linked immunosorbent assay using crude antigens to detect antibody are associated with frequent false-positive and false-negative results and should generally be avoided. For example, Proaño-Narvaez et al.^18^ noted a sensitivity of 41% for ELISA compared with 86% for EITB.^19^ The sensitivity of EITB varies with the form of NCC and specimen. Testing of serum is generally more sensitive than cerebrospinal fluid (CSF) using the EITB assay.^16^ In patients with multiple parenchyma, with ventricular NCC or with SAN, the sensitivity of serum EITB is close to 100%.^16^ However, the sensitivity is poor in patients with a single parenchymal lesion or with only calcifications.^20^

Assays for parasite antigens in CSF, serum, or even urine may also be used to confirm the diagnosis.^21^ Antigen detection assays are not presently available commercially in the United States. They are also thought to be less sensitive than EITB. However, positive results correlate with the number of viable cysticerci. Parasite antigens are commonly detected in both serum and CSF in cases with multiple cysticerci such as SAN and serial measurements may be helpful in the follow-up of complex cases.^22–24^ Antigen detection assays for NCC have been proposed as useful for diagnosis, but these are not presently widely available in North America.

### II. What imaging studies should be used to classify disease?

#### Recommendation.

4. We recommend both a brain MRI and a non-contrast CT scan for classifying patients with newly diagnosed NCC (strong, moderate).

#### Evidence summary.

The pathogenesis, clinical manifestations, prognosis, and management vary depending on the location of the cysticerci and viability of the cysticercus and the associated host response (Tables 1–3).^3,4,8,11,25^ The mildest form of NCC is the patient with an SEL. Lesions in these patients are often cystic. Patients also frequently present with one or more viable cysticerci, which are identified as cystic lesions on neuroimaging studies. A third group of patients presents with parenchymal calcifications. Many of these patients present with chronic epilepsy. A fourth group has one or more cysticerci in the ventricles. They often present with symptoms of obstructive hydrocephalus. Finally, a fifth group has cysticerci in the subarachnoid space and can present with a range of presentations associated with basilar arachnoiditis. Many patients have mixed forms. In this case, they should generally be managed based on the more severe manifestation, with parenchymal disease usually milder whereas ventricular and subarachnoid disease carrying a worse prognosis.

Management depends on careful staging. Neurocysticercosis patients should be classified to determine if they have enhancing parenchymal lesions, viable parenchymal lesions, ventricular disease, SAN, isolated spinal disease, and/or ocular disease (Tables 2 and 3). Patients can have cysts in more than one of the previously mentioned locations. For example, although most cases with NCC have isolated parenchymal lesions, patients with parenchymal lesions may also have additional lesions in parenchyma, subarachnoid space, or ventricles. If possible, MRI should be performed in all cases to look for additional cysticerci. Recent advances in imaging include MRI with 3D volumetric sequencing, such as fast imaging using steady-state acquisition (FIESTA), 3D constructive interference in steady state (3D CISS), or balanced fast field echo (BFFE). These sequences provide enhanced resolution in areas with high T2 signal such as CSF. In cysticercosis, they have enhanced sensitivity for detection of extra-axial cysticerci in the ventricles or subarachnoid spaces.^14,15,26–28^ There is a strong association of basal SAN with asymptomatic involvement of the spine.^29^ Thus, all patients with intracranial subarachnoid disease should also undergo an MRI of the spine.

### III. What additional tests should be performed before initiation of therapy?

#### Recommendations.

5. We suggest screening for latent tuberculosis infection in patients likely to require prolonged corticosteroids (weak, low).
6. We suggest screening or empiric therapy for *S. stercoralis* in patients likely to require prolonged corticosteroids (weak, low).
7. We recommend that all patients with NCC undergo a fundoscopic examination before initiation of anthelminthic therapy (strong, moderate).
8. We suggest that the patient with NCC, who has probably acquired NCC in a non-endemic area have their household members be screened for tapeworm carriage (weak, low). *Remark*: This is a public health issue and can often be addressed by the local health department.

#### Evidence summary.

Management of NCC often involves the use of corticosteroids or other anti-inflammatory therapy. Depending on the anticipated dose and duration of therapy and prior exposures, the patients may be at increased risk for opportunistic infections, including reactivation tuberculosis. Testing for latent infection with *Mycobacterium tuberculosis* is frequently performed in patients with NCC. It is recommended for all patients who will undergo prolonged treatment with corticosteroids.^30,31^ The course of steroids used for many patients with SEL from NCC is often not a clear indication for prophylactic therapy for latent tuberculosis. By contrast, when a month or more of therapy is anticipated, experts believe that patients should be screened for latent tuberculosis and considered for chemoprophylaxis, as would be the case for any subject from endemic areas.

Corticosteroids increase the risk for *S. stercoralis* hyper infection. *S. stercoralis* is co-endemic with *T. solium* in many areas. However, the prevalence is poorly defined in most populations. There is considerable controversy about how best to prevent *Strongyloides* hyper infection in patients from *Strongyloides*-endemic areas, who will be treated with steroids. The rates of hyperinfection are low, and most patients with NCC are treated with albendazole along with steroids, which may successfully treat most patients. Strategies for prevention of strongyloidiasis hyperinfection range from testing all patients for larvae in stool, serologic testing in all patients, stool and/or serologic testing only in symptomatic patients, or empiric treatment with ivermectin. Stool studies require specialized testing for *Strongyloides* such as Baermann’s concentration method. Even when performed, the sensitivity is poor even when testing multiple specimens. Serologic tests for antibody are more sensitive but have lower specificity. Because of difficulty in diagnosis, some authorities recommend empiric treatment with ivermectin rather than depending on imperfect testing.

Fundoscopy is an important part of evaluation of patients with potential hydrocephalus or cerebral edema. By contrast, in parenchymal NCC with small number of cysts or granulomas, fundoscopy is unlikely to detect papilledema. However, fundoscopic examination should be performed to exclude intraocular cysticerci, which occurs in a small proportion of patients. Antiparasitic therapy may lead to blindness in some cases with unsuspected intraocular parasites. An indirect fundoscopic examination may be more sensitive for detection of parasites. Ocular ultrasound examination is an alternative method to screen for ocular involvement.

Patients acquire infection from a tapeworm carrier (usually either the patient with NCC or a close contact). However, there is a prolonged incubation period between infection with NCC and onset of symptoms. Many of the tapeworm carriers who originally transmitted infection may have cleared the intestinal infection or may no longer live near the patient. Presently, stool microscopy is the only available diagnostic test for tapeworms. Stool examination for ova is often negative in tapeworm carriers. Even multiple examinations may not detect the tapeworm carrier. Even when ova are found, the morphology of the ova cannot distinguish *T. solium* from other *Taenia* species. Thus, the yield of microscopy for identification of tapeworm carriers is generally low even in cases with apparent transmission outside of endemic areas. Nevertheless, among patients who apparently acquired infection in the United States, Sorvillo et al.^32^ documented tapeworms in close contacts of 22% of NCC cases. Thus, most authorities would recommend screening for cases acquired outside endemic areas. Newer methods such as antigen detection in stool or detection of tapeworm-stage specific antibodies by immunoblot might improve the usefulness of screening, but these are presently only research techniques and not commercially available at present.

Tapeworm carriers pose a public health risk, especially if they are food handlers. There are also risks of transmission within the household and from mother to child. Thus, identification of a tapeworm carrier is an important public health issue and local public health authorities should be notified of cases of NCC (NCC or tapeworm carriage is reportable in many states and regions, but reporting is not mandated nationally). Public health authorities should be notified of cases and involved in investigation of tapeworm carriers.

### IV. How should antiparasitic and anti-inflammatory therapy be monitored?

#### Recommendations.

9. We recommend that patients treated with albendazole for more than 14 days be monitored for hepatotoxicity and leucopenia (strong, moderate).
10. No additional monitoring is needed for patients receiving combination therapy with albendazole and praziquantel beyond that recommended for albendazole monotherapy (strong, moderate).

#### Evidence summary.

Albendazole is generally poorly absorbed. Absorption can be improved by dosing it with food, especially with fatty meals. The main side effects of albendazole in patients treated with doses of 15 mg/kg/day (up to 1,200 mg/day) or less for 28 days are due to the parasiticidal activity and treatment-induced inflammation, including headaches, seizures, and dizziness Thus, there is a transient increase in the number of seizures after therapy. Hepatoxicity and leucopenia are known side effects of albendazole and are relative contraindications to its continued use. In studies of chronic therapy, mainly for echinococcosis, elevated liver enzymes were seen in up to 16% of cases and required drug discontinuation in 3.8%.^33^ The elevated transaminases normalized in almost all cases when the drug is discontinued promptly. Leucopenia is also noted in up to 10% of cases receiving prolonged therapy, but only requires discontinuation in < 1% of cases. Reversible alopecia may also occur in up to 10% of cases. Most patients tolerate continuous therapy without interruption. Higher doses (30 mg/kg/day) have been used in some cases of subarachnoid cysticercosis, but there are limited data on safety.^34^ Few adverse events were noted with duration of up to 4 weeks. Thus, prolonged or high-dose albendazole can be used when needed (e.g., SAN or giant cysticerci).

Both liver enzymes and complete blood counts should be monitored during the first month in patients receiving albendazole alone or in combination with praziquantel. The consensus of the panel was that patients who will receive albendazole or albendazole plus praziquantel for more than 14 days should be monitored with complete blood counts and liver enzymes during the first month. The optimal frequency of monitoring is unknown, but our panel felt that monitoring laboratory test weekly is adequate. In those receiving prolonged duration of albendazole, liver enzymes should continue to be monitored with the frequency based on clinical indications and tolerance. In the presence of absolute neutropenia or elevation of transaminase more than five times the upper limits of normal, albendazole should be withheld until laboratory tests normalize and alternative approaches considered (e.g., praziquantel or no antihelminthics). This is usually only an issue in prolonged courses of therapy such as those used for subarachnoid disease.

The adverse effects noted with praziquantel depend on the indication, dose, and duration of therapy.^35^ Most adverse effects in patients with NCC are due to its cysticidal activity, including headaches, dizziness, and seizures. Initial dose-ranging studies of praziquantel did not note other significant adverse events with doses of to 50 mg/kg/day for up to 28 days. Doses of up to 100 mg/kg/day for up to 28 days have been used in NCC without additional adverse laboratory adverse events. However, more than 10% of those treated with praziquantel develop gastrointestinal side effects such as nausea, vomiting, or abdominal pain. Allergic reactions, including urticaria and other rashes, are also noted in a small proportion of cases. Thus, patients should be advised about gastrointestinal and allergic reactions.

In two trials of combination therapy using both albendazole and praziquantel in parenchymal NCC, there were no more or different adverse events with combination therapy than with albendazole alone.^36,37^ Just as in monotherapy, liver enzymes and complete blood counts should be monitored.

Antiparasitic drugs can worsen symptoms of NCC by inducing an inflammatory response. Evidence from large case series suggests fewer adverse events in patients treated with antiparasitic drugs and steroids compared with antiparasitic drugs alone. Based on this fact, most authorities recommend using corticosteroids whenever antiparasitic therapy is planned. The doses and duration vary with different forms on NCC (see in the following paragraphs).

Short courses of corticosteroids are usually well tolerated. However, the adverse events profile is well defined, including hyperglycemia and gastritis. Additional risks of prolonged therapy include opportunistic infections, osteopenia, Cushing’s syndrome, aseptic necrosis of joints, altered mood (e.g., depression and psychosis), and skin changes. Thus, prolonged steroid therapy should be used with caution. Best practices are to monitor patients on chronic steroids for these adverse events. Patients on corticosteroids for more than 2 weeks should undergo monitoring for blood sugar. Many authorities place all subjects on an H2 blocker or proton pump inhibitor to prevent gastritis.

Methotrexate has been used as an alternative treatment, especially in those patients who cannot tolerate steroids, or as a steroid-sparing agent during prolonged therapy of SAN.^38^ An initial dose of 7.5 mg weekly can be increased to a maximum of 20 mg weekly. The treatment is generally well tolerated. Hepatotoxicity including cirrhosis, pulmonary complications, and myelosuppression can complicate chronic therapy with methotrexate when used daily for malignancies, but this is rare at doses used for NCC. Other side effects may include gastrointestinal intolerance, stomatitis, macular rash, alopecia, central nervous system problems, and hematologic abnormalities, but are rare with the low doses used in NCC. Patients receiving chronic therapy should receive folate supplementation, but not on the day that methotrexate is given.

## RECOMMENDATIONS FOR THE TREATMENT OF VIABLE INTRAPARENCHYMAL NCC (VPN)

### V. What is the role of antiparasitic drugs in VPN?

#### Recommendations.

11. In patients with untreated hydrocephalus or diffuse cerebral edema, we recommend management of elevated intracranial pressure alone and not antiparasitic treatment (strong, moderate). *Remarks*: Management of patients with diffuse cerebral edema should be anti-inflammatory therapy such as corticosteroids, whereas hydrocephalus usually requires a surgical approach.
12. In the absence of elevated intracranial pressure, we recommend the use of antiparasitic drugs in all patients with VPN (strong, moderate).
13. For patients with one to two viable parenchymal cysticerci, we recommend albendazole monotherapy for 10–14 days compared with either no antiparasitic therapy (strong, high) or combination antiparasitic therapy (weak, low). *Remarks*: The usual dose of albendazole is 15 mg/kg/day divided into two daily doses for 10–14 days with food. We recommend a maximum dose of 1,200 mg/day.
14. We recommend albendazole (15 mg/kg/day) combined with praziquantel (50 mg/kg/day) for 10–14 days rather than albendazole monotherapy for patients with more than two viable parenchymal cysticerci (strong, moderate).
15. We suggest retreatment with antiparasitic therapy for parenchymal cystic lesions persisting for 6 months after the end of the initial course of therapy (weak, low).

#### Evidence summary.

Viable cysts are usually defined based on the radiologic appearance of a cystic lesion with a fluid-filled center. On CT or MRI, the best correlate for a viable organism is a cystic lesion with a hypodense center on CT or T1 images or a hyperintense center on T2 images. By contrast, nonviable lesions lack the cystic component (e.g., lesions with isodense centers or calcified lesions). The natural history of parenchymal NCC includes an asymptomatic period that typically lasts several years, followed by gradual degeneration over a period of at least a year. Although the exact proportion is unknown, many patients with parenchymal NCC go on to develop calcified lesions, a risk factor for chronic epilepsy. A higher number of seizures at baseline, poor adherence to antiepileptic drugs, and development of calcifications are the risk factors for seizure recurrence.

Antiparasitic drugs can worsen cerebral edema and should generally be avoided in patients with increased intracranial pressure from either diffuse cerebral edema (cysticercal encephalitis) or untreated hydrocephalus.^39^ In both cases, antiparasitic drugs can lead to fatal adverse events, such as herniation. Cases with cysticercal encephalitis already have an inflammatory response and management should focus on anti-inflammatory therapy, such as corticosteroids. In cases of hydrocephalus, antiparasitic drugs can also lead to worsening. Thus, increased intracranial pressure should be addressed before initiating antiparasitic therapy.

The use of antiparasitic drugs in cystic NCC was first reported in 1979, yet the role of antiparasitic drugs in cystic lesions remains controversial. Two recent meta-analyses have analyzed data on antiparasitic drug in viable parenchymal cysticercosis.^40,41^ One of these meta-analyses^41^ was limited by not separating the analyses of cystic lesions from enhancing lesions. Most early studies either used historic controls or patients who refused to enroll in trials.^40^ These early studies suggested improved radiologic resolution and clinical prognosis in those treated with either praziquantel or albendazole. However, studies mainly from the United States, also demonstrated a good prognosis in those not treated with antiparasitic drugs.^42^ In the 1990s, several poor-quality, randomized trials were reported in which there was no clear benefit of antiparasitic drugs compared with placebo.^43^ However, there were methodological concerns about these studies.

Despite the large numbers of studies reported on the subject, there are only two high-quality placebo-controlled trials of antiparasitic drugs in viable NCC. Garcia et al.^44^ enrolled 120 patients in a placebo-controlled randomized trial of albendazole 800 mg/day plus dexamethasone 6 mg/day both in divided doses for 10 days compared with placebos for both. Therapy was well tolerated. At 6 months of follow-up, 21/55 in the albendazole group compared with 8/54 in the placebo group demonstrated radiologic resolution on MRI (*P* = 0.007). Carpio et al.^45^ reported a second high-quality, placebo controlled randomized trial of albendazole in cysticercosis. Patients were randomized to albendazole 800 mg/day or placebo for 8 days. Both groups received prednisone 75 mg/day. Separate analyses were done for those with only parenchymal disease and those with both extraparenchymal disease and for those with “active” cysts and those with “transitional” cysts. Among those with active parenchymal cysts, resolution was demonstrated at 6 months in 19/39 (49%) treated with albendazole compared with 8/27 (23%) of those treated with placebo (*P* = 0.021). In both studies, the rate of resolution was much lower in patients with multiple non-enhancing parenchymal cysticerci.

The effect of antiparasitic drugs on seizures in VPN has been difficult to demonstrate. Garcia et al.^44^ noted no seizures between 2 and 30 months of follow-up in 32/57 (56%) patients in the albendazole group and 30/59 (51%) in the placebo group. However, there were 46% fewer seizures in the albendazole and steroid group. The overall reduction in the number of seizures was not significant. However, there was a significant reduction in the numbers of generalized seizures (22 versus 68 *P* = 0.003). Similarly, Carpio noted a higher proportion of patients who were seizure free at 12 months in those receiving albendazole, but the difference was not statistically significant (62% versus 52% *P* = 0.274). However, subgroup analysis has also demonstrated a decrease in the number of recurrent focal seizures with generalization.^46^

Both praziquantel and albendazole have cysticidal activity, but there are limited high-quality data on the relative efficacy of the two drugs in NCC. Treatment with praziquantel has generally been less effective than albendazole. A single randomized trial comparing 2-week courses of these agents did not demonstrate significant differences.^45^ In open-label studies, radiologic response rates with albendazole (generally dosed at 15 mg/kg/day in two daily doses) have tended to be better than those with praziquantel (generally used at doses of 50 mg/kg/day given in three daily doses for 14 days). However, praziquantel has more complex drug interactions, with extensive first pass metabolism induced by drugs such as antiepileptic drugs and corticosteroids, which are often co-administered with antiparasitic drugs. First pass metabolism of praziquantel can be inhibited by cimetidine. Thus, higher drug levels occur with the coadministration of cimetidine and praziquantel, but there are no controlled, clinical data demonstrating the clinical impact of coadministration. A single report and expert opinion also support the use of high doses of praziquantel (such as 100 mg/kg/day). The studies of albendazole demonstrating efficacy have used doses of 15 mg/kg/day divided into two daily doses. Drugs have been continued for 10–14 days. There are limited data on the safety of doses over 1,200 mg/day. Thus, we suggest that doses be limited to 1,200 mg/day.

Three trials have compared the combination of albendazole and praziquantel to albendazole alone for parenchymal NCC. One initial report noted a higher rate of lesion resolution in patients treated with the combination. However, that study was not randomized and did not mask assessments of differences between groups.^47^ In a phase I/II study of albendazole plus placebo compared with albendazole plus praziquantel, Garcia et al.^37^ noted improved radiologic resolution in the combination treatment group (12/16 [75%] with the combination compared with 4/16 [25%] with albendazole alone). Similarly, Garcia et al.^37^ completed a randomized controlled trial of albendazole (15 mg/kg/day up to 800 mg/day), higher dose albendazole (22.5 mg/kg/day), or combination therapy (albendazole 15 mg/kg/day plus praziquantel 50 mg/kg/day) each for 10 days.^36^ Each group was treated with dexamethasone 0.1 mg/kg/day. Among those with three or more cysticerci, on MRI at 180 days of follow-up, resolution of all viable cysts was demonstrated in 13/19 (68%) in the combination group compared with 1/21 (5%) in the standard dose albendazole group and 5/20 (25%) in the high-dose albendazole group (*P* < 0.0001).^36^

Persistence of cystic lesions after chemotherapy is associated with recurrent seizures.^36,48^ Although there are no convincing data that retreatment is better than symptomatic therapy, most experts recommend retreatment of patients with persistent cystic lesions beyond 6–12 months of therapy. Options for retreatment include a second course of albendazole, switching to praziquantel, or using the combination of albendazole and praziquantel. Combination therapy may have superior parasiticidal activity as noted previously.

### VI. What is the role of anti-inflammatory therapy in management of VPN?

#### Recommendation.

16. We recommend adjunctive corticosteroid therapy begun before antiparasitic drugs rather than no adjunctive therapy in all patients treated with antiparasitic therapy (strong, moderate).

#### Evidence summary.

Antiparasitic drugs can worsen symptoms of NCC by inducing an inflammatory response.^44^ Anecdotal evidence suggests fewer adverse events in patients treated with antiparasitic drugs and steroids compared with antiparasitic drugs alone.^49^ Based on this fact, most authorities recommend using corticosteroids whenever antiparasitic therapy is planned. The impact of corticosteroids on lesion resolution and on development of calcifications in VPN is unknown. Trials using corticosteroids along with albendazole have not demonstrated different proportions developing calcifications. A recent trial of enhanced dexamethasone (8 mg/day in three daily doses for 28 days with a 14-day taper compared with 6 mg/day [also three times daily] for 10 days, both receiving albendazole for 10 days at 15 mg/kg and antiepileptic drugs) revealed a significant decrease in partial seizures over the first 21 days as well as over the first 180 days in the enhanced corticosteroid arm.^50^ There was no significant difference in cyst resolution or other side effects between the arms. However, the study was underpowered because of slow enrollment. Although the optimal dose of corticosteroids has not been defined, this study suggests that higher doses may be preferable when patients are treated for VPN.

### VII. What is the role of antiepileptic drugs in VPN?

#### Recommendations.

17. We recommend antiepileptic drugs in all NCC patients with seizures (strong, low).
18. In patients with few seizures before antiparasitic therapy, resolution of the cystic lesion on imaging studies, and no seizures for 24 consecutive months, we suggest that tapering off and stopping antiepileptic drugs be considered (weak, moderate).
19. In the absence of controlled data, the choice of antiepileptic drugs should be guided by local availability, cost, drug interactions, and potential side effects (fact, no grade).

#### Evidence summary.

Although there are no controlled trials, antiepileptic drugs appear to be as effective at controlling seizures in patients with parenchymal NCC as they are in other seizure disorders. There is also indirect evidence of effectiveness. For example, poor adherence to antiepileptic drugs is a major risk factor for seizure recurrence.^44^ Antiepileptic drugs should be used in patients with VPN and seizures.^44^

There are no controlled data comparing efficacy of different antiepileptic drugs in patients with viable parenchymal cysticercosis. Phenytoin, carbamazepine, and phenobarbital have been used in many cases. Besides efficacy, other considerations that need to be considered include drug interactions with antiparasitic agents and corticosteroids, which are especially problematic for phenobarbital. Although any antiepileptic drug can be used, it may be better to avoid phenobarbital with antiparasitic therapy, because of high rates of drug interactions. Newer agents such as levetiracetam with fewer drug interactions may be preferable to older agents.

There are limited data on optimal duration of antiepileptic drugs (AEDs) in patients with viable parenchymal cysticerci.^51^ Anecdotal reports suggest that antiepileptic drugs can eventually be effectively tapered and discontinued in patients with resolution of the parasitic cysts. In prospective studies, seizure recurrences have been noted in those who discontinued antiepileptic drugs after being seizure free for a least 1 year. Guidelines for management of idiopathic seizures suggest continuations of antiepileptic drugs for at least 24 months. Risk factors for seizure recurrence include the number of seizures before treatment and development of calcified lesions.^48,52^ Recurrence of seizures is unusual in patients with CT resolution without the development of calcifications and no subsequent seizures for more than 12 months.^48,52^

### VIII. What follow-up is recommended after initial antiparasitic therapy for patients with VPN?

#### Recommendation.

20. We suggest that MRI be repeated at least every 6 months until resolution of the cystic component (strong, low).

#### Evidence summary.

Patients should be followed clinically for seizure recurrence and optimization of antiepileptic drugs as recommended for other patients with seizures. The first clinical follow-up after initial diagnosis should be performed at 2–4 weeks to determine if the patient has developed any recurrent seizures or new or worsening symptoms/signs. New or worsening symptoms should prompt re-imaging. Monitoring of the antiparasitic response should mainly involve serial imaging studies. At a minimum, an MRI should be performed at least every 6 months until resolution of the cystic lesion, as we suggest retreatment in those with persistent lesions. This recommendation of frequency of follow-up scans is largely based on expert opinion. However, data from controlled trials of antiparasitic therapy have demonstrated that most cystic lesions have resolved by 6 months of follow-up.^44,45^ Some recommend earlier imaging to look for an initial response to therapy or with clinical worsening. A CT scan should be performed before consideration of stopping antiepileptic drugs to determine if calcifications have developed.

## RECOMMENDATION FOR THE TREATMENT OF DEGENERATING INTRAPARENCHYMAL NCC INCLUDING PATIENTS WITH SEL DUE TO NCC (SEL, ALSO TERMED SOLITARY CYSTICERCUS GRANULOMA)

### IX What should be the initial approach to the patient with multiple enhancing lesions from NCC?

#### Recommendation.

21. We recommend that patients with multiple enhancing lesions and seizures be initially treated with antiepileptic drugs, antiparasitic therapy, and corticosteroids as outlined in the section on viable parenchymal cysticerci (weak, moderate).

#### Evidence summary.

Patients with one or, perhaps, two enhancing lesions have a better prognosis than those with multiple cysts.^53^ Clinical trials of therapy for patients with viable cysticerci often included patients with multiple enhancing cysticerci.^44,45^ Thus, recommendations for viable cysticerci can also apply to these patients. By contrast, the data on optional treatment choice for patients with SEL are significantly different and has been studied separately.

### X. What is the role of antiepileptic medications in patients with SEL from cysticercosis with seizures?

#### Recommendations.

22. We recommend antiepileptic drugs for all patients with SEL and seizures (strong, moderate).
23. In the absence of controlled data, the choice of antiepileptic drugs can be guided by local availability, cost, drug interactions, and potential side effects (fact, no grade).
24. In patients who have been seizure free for 6 months, we suggest tapering off and stopping antiepileptic drugs after resolution of the lesion in patients with SEL without risk factors for recurrent seizures (weak, moderate). *Remark*: Risk factors for recurrent seizures include residual cystic lesions or calcifications on neuroimaging studies, breakthrough seizures, or more than two seizures.

#### Evidence summary.

In one prospective cohort study of 185 patients,^54^ most did not develop recurrent seizures. A minority of patients (16.2%) developed a seizure 1 week or more after the initial seizure while on AEDs. With long-term follow-up of 24 to 125 months, 28 patients (15.1%) with SEL developed recurrence of seizures after withdrawal of AEDs.

Symptomatic therapy in the form of antiepileptic drugs is recommended for all patients with SEL and seizures. Those who present with headache alone (about 7%) do not need antiepileptic drugs.^12^ The goals of therapy are to prevent subsequent seizures in those presenting with seizures. The impact of AEDs on the natural history of seizures is not known. Because 16.2% developed seizures in spite of antiepileptic therapy, it is likely that this number would be higher if AEDs were not prescribed.

There is limited evidence for the superiority of a particular AED. We suggest that the choice of antiepileptic drugs be guided by local availability, cost, drug interactions, and side effects. Monotherapy with phenytoin or carbamazepine-controlled seizures in 86.5% of patients in one study involving 185 patients with SEL,^54^ A single, open-labeled comparative trial of clobazam versus phenytoin in patients with SEL suggested that the former might be more effective. However, clobazam has not been widely used. In contrast, carbamazepine and phenytoin appear to be used most often, largely because of availability and cost considerations in *T. solium*–endemic regions.^55^ Both are potent hepatic P_450_ enzyme inducers, and the antihelmithic drugs praziquantel and albendazole are metabolized by the hepatic P_450_ enzyme system. In pharmacokinetic studies, phenytoin and carbamazepine decrease the area under curves of praziquantel and to a lesser extent, albendazole.^44,56,57^ The clinical significance of the pharmacokinetic interaction in context of a solitary cysticercus granuloma is unknown. One potential way of circumventing this interaction is to administer a nonenzyme-inducing AED (e.g., levetriacetam) at least for the period of time for which antihelminthic treatment is being administered. This treatment strategy, however, has not been tested in any controlled trial.

An important management issue resolves around the duration for which AED treatment is given. At least three trials compared short-term (6 months) with slightly longer term (12–24 months) AED treatment.^58–60^ The trials found no benefit in seizure control with longer duration AEDs in those people in whom the solitary cysticercus granuloma had completely resolved. The studies also revealed that the risk of recurrent seizures remained high in people in whom the lesion resolved but resulted in a calcific residue visible on CT scan. The increased risk of seizures in those who developed calcification appeared to be offset by using a longer duration of AED treatment (12–24 months).^55^ Hence, people with solitary cysticercus granuloma, in which the granuloma leaves behind a calcific residue, should receive a longer duration AED. How long should AEDs be advocated in these circumstances and when AEDs can be safely discontinued remains unsettled, and AEDs should probably be managed according to the guidelines for chronic epilepsy.

### XI. What is the role of antiparasitic drugs in patients with SEL?

#### Recommendation.

25. We suggest albendazole therapy rather than no antiparasitic therapy for all patients with SEL (weak, moderate). *Remarks*: Albendazole (15 mg/kg/day in twice daily doses up for 1–2 weeks) with meals.

#### Evidence summary.

The role of antiparasitic drugs in patients with SEL has been controversial. Initial treatment studies with praziquantel and later albendazole demonstrated that patients treated with antiparasitic drugs had few adverse events and a benign course.^61,62^ However, the same pattern was noted in those treated with only antiepileptic drugs.^54,63^ In a meta-analysis, Otte and colleagues identified 10 randomized controlled trials of antiparasitic treatment of patients with one to two enhancing cysticerci, involving 765 subjects.^5,9–18^ There was considerable heterogeneity in the studies regarding the duration of albendazole (3–28 days), frequency of follow-up, and radiologic responses. A single study compared single-day treatment with praziquantel to placebo and demonstrated a higher rate of radiologic resolution at 3 months.^64^ Most lesions in the placebo arm demonstrate resolution with symptomatic therapy alone within a year. However, the radiologic resolution tends to be more rapid in patients treated with albendazole (usually with corticosteroids). This meta-analysis concluded that albendazole treatment modestly accelerates resolution of lesions and seizure freedom in patients with SEL. Six trials reported radiologic resolution at 6 months resolution, which was noted in 142/212 (67%) treated with albendazole versus 80/168 (48%) treated with placebo, *P* = 0.04.^58,65–69^ However, there was considerable heterogeneity between trials. Seven studies examined the number of subjects that remain free of seizures, but the length of follow-up was variable. In the five studies reporting follow-up at 6 months, 180/199 (90%) were seizure free in the albendazole arm compared with 137/165 (83%) treated with placebo.^58,66,68–70^ A single study reported 12 month follow-up and reported no significant difference in those who were free of seizures.^67^ Overall, there was no significant difference in the frequency of calcifications at the end of follow-up, a surrogate marker for chronic epilepsy.

A single trial compared treatment with albendazole alone to albendazole combined with praziquantel.^71^ There were trends toward more rapid resolution and fewer residual calcifications in the combination arm, but the results were not statistically significant.

The duration of albendazole therapy for SEL in different studies has ranged from 3 to 30 days. In comparative studies, no significant differences were demonstrated comparing 7 and 28 days, 7 and 14 days, or 2 and 15 days (although there was a trend toward a better response in the latter).^72^ Based on that data, our panel suggests that therapy should be administered for 7–14 days.

### XII. What is the role of anti-inflammatory therapy in SEL?

#### Recommendation.

26. We recommend that patients with SEL treated with antiparasitic drugs should also be treated with corticosteroids initiated before antiparasitic therapy (strong, moderate).

#### Evidence summary.

In the meta-analysis of Otte et al.,^73^ randomized studies of corticosteroids alone (i.e., not concomitantly with antihelminthic drugs) in patients with SEL did not demonstrate a significant impact on lesion resolution, seizure recurrence, or development of calcifications. However, that analysis misinterpreted data from one study.^74^ The group assignments were different in the abstract (suggesting worse with steroids) than in the body of the article and conclusions (steroids were better).^74^ If that study was analyzed correctly, the results would have been statistically significant. A subsequent meta-analysis using network analysis noted that the combination of albendazole plus corticosteroids had the optimal effect on lesion resolution and recurrent seizures.^75^

### XIII. How should patients with SEL be followed?

#### Recommendation.

27. We suggest that MRI be repeated at least every 6 months until resolution of cystic lesions for patients with SEL (weak, low).

#### Evidence summary.

There are limited data on optimal follow-up of patients with SEL due to NCC. Most controlled trials have followed the patients at 3–6 months after therapy. Data suggest that imaging studies often normalize and antiepileptic drugs can often be discontinued after 6 months of therapy. Based on this, follow-up at 6 months appears to be important.

## RECOMMENDATIONS FOR THE TREATMENT OF CALCIFIED PARENCHYMAL NEUROCYSTICERCOSIS (CPN)

### XIV. What should the initial approach be to patients with calcified lesions suggestive of CPN?

#### Recommendation.

28. We suggest brain MRI in patients with seizures or hydrocephalus and only CPN on CT (weak, low).

#### Evidence summary.

The extent of evaluation required for patients with CPN depends on symptoms. Asymptomatic subjects without evidence of viable cysts do not require additional evaluation. By contrast, patients with seizures should be studied with MRI to exclude co-existing viable cysticerci, especially in the posterior fossa. Patients with symptoms of increased intracranial pressure should be studied by MRI looking for subarachnoid or ventricular disease.

The calcifications are best visualized by non-contrast CT examination (most sensitive), but can also be identified by MRI using gradient echo techniques (generally less sensitive). Typical calcifications are usually small (e.g., 1–4 mm), dense, and round but at times are large and irregular in shape. The number of calcifications varies from one to hundreds but most patients have one to two calcifications. Calcified lesions may not be diagnostic but in endemic areas are highly suggestive of NCC. The scolex embedded within the calcification can sometimes be visualized using MRI gradient spin echo imaging in some cases. Perilesional edema and the absence of viable cysts are best visualized by MRI using FLAIR or T2 sequences. Calcifications appear as voids or black regions by MRI. At present, the management of patients with transient perilesional edema (caused by calcifications) is symptomatic and similar to other patients with calcifications. Thus, repeated imaging is only recommended in the setting of new or worsening symptoms. Serologic tests including EITB are frequently negative in patients with only calcifications, and parasite antigens in the blood or CSF are not generally detected.

### XV. What is the role of antiparasitic drugs, antiepileptic drugs, and anti-inflammatory medications in the management of patients with CPN?

#### Recommendations.

29. We recommend symptomatic therapy alone instead of antiparasitic drugs in patients with calcified parenchymal lesions (strong, moderate).
30. We suggest that corticosteroids not be routinely used in patients with isolated CPN and perilesional edema (weak, low).

#### Evidence summary.

Calcified lesions do not contain viable parasites. Thus, there is no reason to use antiparasitic therapy. Before widespread use of CT scanning, calcifications were primarily identified and characterized by gross examination at autopsy as hard or chalky nodules in the brain.^76^ Microscopic descriptions noted varying amounts of degenerated membranes, calcareous corpuscles, and areas of amorphous calcifications surrounded by a collagenous capsule with little or no surrounding or intralesional inflammation. This contrasts with viable organisms and exuberant inflammation that is commonly associated with degeneration cysts and led to the suggestion that calcified granulomas are “inactive” and play little or no part in the pathophysiology of the disease.^77^ However, some calcified granulomas are foci of the seizure activity.^78,79^

There are limited data on cessation of antiepileptic drugs in patients with calcified NCC. Existing guidelines for the treatment and prevention of epilepsy including choice of agents and doses and cessation of antiseizure medications should be followed. In prospective studies, at least 36% of patients developed recurrent seizures.^80^ Risk factors for recurrences have included the number of prior seizures and seizure-free interval. Thus, those with few prior seizures and a long seizure-free period (e.g., 2 years of good control) may be candidates for tapering off and discontinuing antiepileptic drugs.

Approximately 30–50% of the time, transient episodes of perilesional edema develop around calcified foci that are foci of seizure activation when symptoms first appear.^81^ Perilesional edema, which is most intense immediately surrounding the calcification, is best visualized using the FLAIR MRI technique; marked enhancement is almost always present as well. In an urban population in Lima with a history of seizures and only calcifications, 50% with recurring seizures had perilesional edema. These data indicate that this phenomenon is common and likely a major cause of seizures in endemic rural populations where the prevalence of calcifications is high.^82^ The natural history is incompletely known, but repeated episodes are common, sometimes reoccurring multiple times over decades.^80^ Perilesional edema regresses without treatment in 3–6 weeks.

It is likely that perilesional edema is due to an immune mediated inflammatory response to antigens present in the calcified granuloma. This is supported by the persistence of microglial activation around calcifications during and following episodes despite the early resolution of edema.^83^ Further evidence of an immune-regulated process is suggested by the apparent induction or reactivation of perilesional edema after abrupt corticosteroid withdrawal.^84^ There appear to be functional and histopathological differences among calcified granulomas. A subset demonstrate enhancement, which is due to abnormal leakage of serum components by the blood–brain barrier^85^ mostly likely caused by persisting perivascular inflammation and gliosis. An inflammatory cause of perilesional edema episodes is further supported by the presence of exuberant capsular and intralesional inflammation in calcified granulomas surgically removed from two patients experiencing seizures and perilesional edema episodes.^3,86,87^ Both the presence of a scolex within the calcification^88^ gliosis around a calcification are associated with increased seizure activity.^89^

There are no specific proven measures for treatment or prevention of symptoms related to calcifications with or without associated perilesional edema. High-dose corticosteroids are commonly used to control edema in other brain conditions. Because perilesional edema appears to be inflammatory, the use of chronic immunosuppressive agents might be expected to be useful. Immunosuppressive drugs such as methotrexate have also been tried. For example, the administration of methotrexate appeared to prevent further incapacitating episodes of perilesional edema in one patient for about 8 years.^38^ However, the use of corticosteroids and other immunosuppressive drugs to control symptoms related to perilesional edema episodes is clouded by the apparent initiation or exacerbation of perilesional edema episodes after abrupt corticosteroid withdrawal.^84^ Thus, at present, there is not sufficient evidence to determine whether the benefits of these drugs outweigh the risks.

### XVI. Is there a role for surgical therapy in refractory cases?

#### Recommendation.

31. In patients with refractory epilepsy and CPN, we suggest evaluation for surgical removal of seizure foci (weak, low).

#### Evidence summary.

Calcified NCC has been associated with refractory epilepsy, although this is uncommon. In some cases, the focus can be mapped to the calcification. In others, the calcification may be associated with hippocampal sclerosis and mesial temporal lobe epilepsy. There are case series of surgical resolution of epilepsy in calcified NCC when the seizure focus is removed.^90,91^ Thus, mapping of the seizure foci followed by surgical therapy should be considered in refractory cases.

## RECOMMENDATIONS FOR THE TREATMENT OF INTRAVENTRICULAR NEUROCYSTICERCOSIS (IVN)

### XVII. How are extra-parenchymal cysts best identified?

#### Recommendation.

32. We recommend MRI with 3D volumetric sequencing to identify intraventricular and subarachnoid cysticerci in patients with hydrocephalus and suspected NCC (strong, moderate).

#### Evidence summary.

In most case series, 10–20% of patients with NCC present with cysticerci in the ventricles.^11,92^ These cysticerci are typically asymptomatic for years before the development of symptoms. Most cases present clinically when the cysticerci obstruct CSF flow causing hydrocephalus. The cysticerci can be found in any of the ventricles. Obstruction typically occurs when the cysticerci lodge in an area of narrowing at the outflow of the ventricle such as the foramen of Monro, entrance to the aqueduct of Sylvius, or foramenae of Luschka or Magendie. Older series noted most of the ventricular cysticerci in the fourth ventricle, but this may have reflected more severe disease when the cysticerci are in that location. In most cases, the cysticerci cause symptoms while still viable.^93^ Because viable cysticerci have thin walls with cyst fluid isodense with CSF, they may be difficult to detect on imaging studies. Computed tomography scanning usually only shows evidence of obstructive hydrocephalus and/or distortion of the shape of the involved ventricle. Magnetic resonance imaging and CT ventriculography are more sensitive. However, MRI with 3D volumetric sequencing (FIESTA, 3D CISS, or BFFE)^14,26,94,95^ are considered to be the best methods of identifying intraventricular cysts. These patients may also have parenchymal cysticerci, which can be associated with parenchymal inflammation and seizures.

### XVIII. What is the optimal approach to management of IVN in the lateral and third ventricles?

#### Recommendation.

33. When possible, we recommend removal of the cysticerci by minimally invasive, neuroendoscopy over other surgical or medical approaches for cysticerci of the lateral and third ventricles (strong, moderate). *Remark*: Most experts recommend that antiparasitic drugs not be used preoperatively because such treatment could result in disruption of parasite integrity and an inflammatory response that could prevent successful cyst removal.

#### Evidence summary.

Ventricular neurocysticercosis presents with symptoms or signs of raised intracranial pressure. Symptoms may include headaches, nausea or vomiting, altered mental status, visual changes, or dizziness. The onset varies and can be abrupt (because of acute obstruction), intermittent, or gradual. Cysticerci may form a ball valve in the foramina that may come and go with position changes. This is especially common for cysticerci in the lateral ventricles. Cysticerci in the fourth ventricle have been associated with acute obstructive hydrocephalus that can lead to drop attacks. (Bruns syndrome).^96^ Ventricular disease is prone to a high case fatality rate and requires careful management.

There are no high-quality data on optimal management of ventricular neurocysticercosis. All patients with obstructive hydrocephalus should be treated by either cyst removal or CSF diversion. Before the 1990s, most patients were managed by surgical excision of the cysticerci from the ventricles.^97,98^ The cure rate was high, but this approach was associated with significant operative morbidity and some mortality, especially for cysticerci in the third ventricle. Furthermore, cases in which the cysticerci were adhered to ependymal or associated with significant periventricular inflammation (as defined by enhancement on imaging studies or marked CSF pleocytosis) could not be readily removed without high rates of surgical complications.^98^

The development of minimally invasive surgical approaches via neuroendoscopy has been increasingly used in neurosurgery. Early reports noted high cure rates and minimal morbidity when lateral and third ventricular cysts were removed by neuroendoscopic approaches.^11,99–104^ A number of case series have now been reported with this approach and outcomes have been generally favorable. Initial concerns were raised about the consequences of rupture of the cysticerci, which has been a frequent occurrence.^99^ To date, there is no convincing evidence of adverse outcomes with cyst rupture. Only retrospective data are available comparing management strategies. However, in case series, outcomes were better with endoscopic removal than open procedures.^11,103^

There are, however, contraindications to endoscopic cyst removal. Inflamed or degenerating cysticerci are frequently adherent to the ventricular walls and ependyma. As is the cases for open removal, attempted removal of adherent cysts is associated with a high risk of hemorrhage and neurologic sequelae. Thus, inflamed cysticerci are a contraindication to neurosurgical removal, regardless of whether they are approached by neuroendoscopy or microdissection. Unlike hydatid disease, cysticerci do not produce daughter cysts in the human host. However, antiparasitic drugs may induce cyst inflammation making cyst removal more difficult. For those reasons, pre-operative antiparasitic therapy should generally be avoided.

### XIX. What is the optimal surgical approach to management of IVN in the fourth ventricle?

#### Recommendation.

34. In cases in which surgical removal of fourth ventricular cysticerci is possible, we recommend surgical removal rather than medical therapy and/or shunt surgery (strong, moderate).

#### Evidence summary.

There are more limited data on endoscopic approaches to cysticerci in the fourth ventricle. There are reports of excision of fourth ventricular cysts via the lateral and third ventricles and aqueduct.^99,103–106^ Case series note excellent results. However, concerns remain regarding the safety of this procedure because the approach requires removal through the aqueduct, and should only be attempted by an experienced neuroendoscopist. Cysticerci in the fourth ventricle can also be approached through a suboccipital craniotomy with cyst excision by either microsurgical dissection or by neuroendoscopy. As is the case with any surgery, attempted removal of adherent cysticerci is associated with a high rate of complications.

### XX. What is the optimal approach to adherent IVN?

#### Recommendation.

35. We suggest shunt surgery for hydrocephalus rather than cyst removal when surgical removal is technically difficult (weak, low). *Remark*: Attempted removal of inflamed or adherence ventricular cysticerci is associated with increased risk of complications.

#### Evidence summary.

Medical treatment alone with antiparasitic drugs and corticosteroids has been proposed.^107,108^ However, most of the cases described were actually treated with a shunt procedure before medical therapy, and there are limited data on the safety and efficacy of medical therapy. Medical therapy alone has been associated with poor outcomes in some series.^11^

In the 1990s, investigators reported management of IVN with CSF diversion alone (typically via a ventriculoperitoneal shunt). However, most cases were complicated by shunt failure.^11,93,109^ In comparative studies, neurologic outcome with shunting alone was worse than with cyst removal either endoscopically or by open procedures. Thus, shunting should be regarded as an alternative strategy when cyst removal is not possible (e.g., with adherent or inflamed cysticerci). Three approaches were used to try to prevent shunt failure, including low-flow shunts, adjunctive corticosteroids, and antiparasitic drugs. There is no good evidence to support any of these approaches. Sotelo et al.^110^ reported using a low-flow shunt and noted lower rates of shunt failure. However, subsequent studies have noted inconsistent control of hydrocephalus with these shunts and they are not widely available.^111^

### XXI. Does medical therapy as an adjunct to procedures or as primary therapy have an impact on outcome in treating patients with IVN?

#### Recommendations.

36. We recommend corticosteroids to decrease brain edema in the perioperative period (fact, no grade).
37. We suggest antiparasitic drugs with corticosteroid therapy after shunt insertion to decrease subsequent shunt failure in patients in whom surgical removal of isolated intraventricular cysts is not possible (weak, low) but neither after successful removal of intraventricular cysts (weak, low). *Remark*: Note that intraventricular cysts may be accompanied by other lesions with indications for antiparasitic therapy.

#### Evidence summary.

There are no high-quality data on the use of corticosteroids in ventricular NCC. Corticosteroids are often administered as an adjunct before, during the surgery, and in the postoperative period to reduce inflammation and brain edema. Although there are no good data on the efficacy for these indications in NCC, they are well established in other neurologic diseases. There is anecdotal experience using high-dose corticosteroids (e.g., dexamethasone 8–24 mg/day in divided doses) to stabilize patients before definitive surgical therapy.

Treatment with shunts without antiparasitic drugs had a high rate of shunt failure (approximately 60%). Suastegui Roman et al.^112^ treated patients who had received ventriculoperitoneal shunts for IVN with prednisone 50 mg 3 days/week and were also noted to have few shunt failures. However, these data have not been confirmed in other studies, and the risks of chronic corticosteroids are significant. Lower rates of shunt failure were noted in those who received antiparasitic drugs and corticosteroids.^93,107^ None of these studies included randomized controls. However, given the significant morbidity associated with shunt failure, we suggest that patients undergoing shunting also be treated with antiparasitic drugs and steroids.

Some authorities have recommended that antiparasitic drugs be used after successful removal of ventricular cysts.^113^ This recommendation came from the time in which earlier generation CT and MRI were used for imaging. Recent data have noted that when cysts are successfully removed by either endoscopy or open surgery, if imaging studies are negative, outcomes are excellent without further antiparasitic therapy (T. Nash, unpublished data).

## RECOMMENDATIONS FOR SUBARACHNOID NEUROCYSTICERCOSIS (SAN)

### XXII. What is the role of medical therapy in SAN in the basilar cisterns or Sylvian fissures?

#### Recommendations.

38. We recommend that patients with subarachnoid cysts should be treated with antiparasitic drugs (strong, low).
39. We suggest that antiparasitic therapy be continued until there is radiologic resolution of viable cysticerci on MRI and resolution of other evidence of cysticerci (weak, low). Responses often require prolonged therapy, which can last for more than a year.
40. We recommend anti-inflammatory therapy (such as high-dose corticosteroids) for SAN initiated before antiparasitic drugs (strong, moderate).
41. We suggest that methotrexate be considered as a steroid-sparing agent in patients requiring prolonged courses of anti-inflammatory therapy (weak, low).

#### Evidence summary.

Cysts of *T. solium* that lodge or develop within the subarachnoid spaces of the brain frequently cause serious disease with considerable morbidity (SAN). Structurally normal cysts commonly lodge in the subarachnoid spaces within fissures abutting on the surface of the brain of the convexities of cerebral hemispheres. These cases present with a benign clinical course similar to parenchymal cysts and should be managed similarly.^3,114^ When located in the basal cisterns of the subarachnoid space or Sylvian fissure, the manifestations can be severe. There is almost always accompanying host inflammatory responses to the parasite resulting in meningitis, edema, and scarring, which are responsible for complications, including communicating hydrocephalus, focal neurological symptoms, nerve entrapments, cerebrovascular complications, including lacunar infarctions, thrombotic strokes, and hemorrhages. Intracranial hypertension is a cause of morbidity and mortality^115,116^ in SAN. It is usually due to hydrocephalus, which is a common complication usually requiring shunt placement.^117–119^

Vascular complications may be the initial presentation with strokes occurring in 4–12% of patients^120,121^ but may occur at any time during the course of disease. Perivascular inflammation can result in endarteritis, inflammatory aneurysms, and thrombosis.^120–124^ Most cases of cerebral infarction associated with NCC involve occlusive arteritis in small perforating vessels, resulting in lacunar infarcts.^125,126^ Large vessel strokes and hemorrhage occur but are less common.^127,128^

Other manifestations of SAN depend on the location and extent of infection. These include cognitive dysfunction, psychiatric disease, and Parkinson’s-like syndromes.^129–132^ Papilledema, frequently observed in patients with hydrocephalus, may lead to optic nerve damage and atrophy resulting in visual loss or impairment.^133^ Cranial nerves can become compromised leading to isolated neurologic deficits.^116,134,135^

There are no randomized or well-controlled studies on antiparasitic treatment in SAN. Before the availability of antiparasitic drugs, patients with SAN had a very poor prognosis. For example, Sotelo and Marin^136^ documented a 50% mortality rate in patients treated with ventriculoperitoneal shunting alone. Several early small series reported successful treatment with short courses of albendazole at doses used in parenchymal disease.^107,109,137^ However, these reports have not been confirmed, with treatment failure and the requirement for multiple courses of therapy noted in most patients. Most experts have concluded that this form of NCC is poorly responsive to cysticidal agents at doses and durations developed for parenchymal disease. A study of 33 patients with giant cysts found that patients had a good response to repeated courses of albendazole and/or praziquantel.^115^ Most cases failed a single course of therapy, but eventually responded to repeated courses of albendazole or praziquantel. Interestingly, no deaths were reported in that series, in sharp contrast to historical controls not treated with antiparasitic drugs.^136^ Thus, most experts have concluded that there are benefits of cysticidal therapy, but that more intensive therapy may be needed that is traditionally used for parenchymal disease.

Alternative treatment regimens have been tried in SAN. Some investigators have noted good outcomes with longer courses of treatment compared with parenchymal disease.^116,118^ High doses of albendazole (30 mg/kg/day) in short courses have demonstrated better cysticidal activity than traditional doses, but the safety of this approach is not well established.^34,138^ Based on the improved response of patients with multiple parenchymal cysticerci to the combination of albendazole (15 mg/kg/day) and concurrent praziquantel (50 mg/kg/day), some experts have begun to use this combination in patients with subarachnoid disease. However, at present, none of these data have been published. Corticosteroids are universally used to prevent damaging effects of ongoing and cysticidal-induced host inflammation, but the manner of use is practitioner dependent. The duration and dosing of cysticidal drugs and steroids or other immunosuppressive drugs have not been studied. Our expert panel suggests that the duration of therapy should be individualized. Our practice is to continue treatment until there is resolution of cystic lesions on neuroimaging studies. In addition, steroids should be continued until resolution of other evidence of viable cysticerci including CSF abnormalities when present and resolution of antigens in CSF and/or serum when detected.

Inflammation plays a key role in the pathogenesis of SAN. Nearly all of the complications of SAN are the result of the inflammatory reaction to parasite antigens. Complications include communicating hydrocephalus, meningitis, and vasculitis. Thus, concomitant corticosteroid therapy is essential to avoid complications due to the ensuing inflammation in the subarachnoid space especially after the administration of antiparasitic agents with a careful tapering schedule to avoid cerebrovascular complications and hydrocephalus.^127,139^ It is usually prudent to stabilize the patient clinically before initiating antiparasitic therapy, which includes administration of high-dose corticosteroids to suppress on-going as well as antiparasitic drug-induced inflammation. Anti-inflammatory treatment is indicated for the duration of antiparasitic cysticidal therapy, which often is continued for months. Because high-dose corticosteroids are associated with serious complications, some authorities recommend using methotrexate as a steroid-sparing agent. Close follow-up of the patient is essential while corticosteroids are being tapered.

Corticosteroids are critical in the setting of intracranial hypertension to stabilize the patient. Doses are not well standardized, but may include dexamethasone, 0.2 mg/kg/day. Untreated hydrocephalus is a contraindication to antiparasitic therapy. Hydrocephalus usually requires shunt placement or cyst removal.

Endpoints for therapy have not been established. The MRI should be followed and examined for regression of the cystic lesions and improvement of enhancement. Enhancement may not completely disappear and the subarachnoid space may remain distorted, presumably because of scarring, presenting a challenge for the clinician deciding on a course of therapy. Cerebrospinal fluid analysis may be helpful if lumbar puncture can be performed safely. Cellularity and hypoglycorrhachia should normalize, but in patients with shunts, the protein may remain elevated. Antigen detection of the secreted metacestode antigens using a monoclonal antibody-based test is a useful adjunctive tool for following SAN patients undergoing treatment.^22–24,114,140–142^ Whereas antigen detection assays are presently commercially available in Europe, standardized assays are not commercially available in the United States. Normalization of CSF cellularity and glucose, absence of cystic lesions on MRI, and negative antigen assays should be an endpoint for ending therapy. Long-term follow-up is required. Subarachnoid NCC is a slowly developing infection and successful therapy may require years of treatment.

### XXIII. What is the role of neurosurgery in SAN?

#### Recommendation.

42. We recommend that patients with hydrocephalus from SAN be treated with shunt surgery in addition to medical therapy (strong, low).
43. We suggest that some patients may benefit from surgical debulking over shunt surgery alone (weak, low).

#### Evidence summary.

Theoretically, surgical removal of parasite mass would be expected to shorten the course of treatment and decrease provoked inflammation. However, because there is a risk of surgery and bleeding due to adherent cysts, the role for debulking in SAN is controversial. Several groups have described endoscopic removal of cysts from SAN via a third ventriculostomy.^103^ When successful, this approach may lead to faster resolution of the cysticerci and decreased antigen-stimulated inflammation. However, this approach is not always possible and attempts to remove adherent cysts in the basilar cisterns can be catastrophic.^143^

## RECOMMENDATIONS FOR SPINAL NEUROCYSTICERCOSIS (SN)

### XXIV. How is SN best treated?

#### Recommendations.

44. We recommend corticosteroid treatment of patients with SN with evidence of spinal cord dysfunction (e.g., paraparesis or incontinence) or as adjunctive therapy along with antiparasitic therapy (strong, moderate).
45. We suggest that both medical (antiparasitic drugs plus anti-inflammatory drugs) and surgical approaches be considered for SN (weak, low). *Practice Statement*: There are anecdotes of good responses of SN to medical and/or surgical therapy. However, there are no good data supporting one approach over the other. We suggest that management of SN should be individualized based on symptoms, location of the cysticerci, degree of arachnoiditis, and surgical experience. Recommendations for antiparasitic drugs, reimaging, and follow-up of SAN should also be considered for subarachnoid SN.

#### Evidence summary.

Corticosteroids are recommended in all patients with evidence of cord dysfunction. Doses and duration should follow guidelines for management of cord dysfunction. In contrast to cases of spinal epidural abscess, cord compromise is frequently due to edema surrounding cysticerci and is not necessarily a surgical emergency. Similar to subarachnoid disease of the brain, cysticidal treatment can worsen inflammation and exacerbate neurologic symptoms. Corticosteroids should be used concomitantly with antiparasitic agents.

There are no good data on optimal management of SN. Intramedullary SN has traditionally been treated with laminectomy and subsequent myelotomy. There have been increasing numbers of reports of treatment with cysticidal drugs with complete recovery and disappearance of lesions on neuroimaging. A recent review suggested that the outcome of intramedullary cysticercosis may be better for medically treated patients than for surgically treated patients.^144^ A trial with cysticidal drugs in suspected cases is warranted to confirm the diagnosis by documenting the resolution of the lesion on follow-up imaging studies. If antiparasitic drugs are used, they should be given together with sufficient corticosteroids to reduce the risk of transient clinical deterioration secondary to worsening inflammation and edema.

For those patients with subarachnoid disease of the spine and concurrent basilar SAN, medical therapy should be undertaken as outlined in the subarachnoid section. Corticosteroids are critical in patients with symptomatic extramedullary spinal disease. As is the case for cerebral subarachnoid disease, doses are not well standardized, but may include dexamethasone 0.2 mg/kg/day with albendazole 15 mg/kg/day. The clinical picture along with the MRI of the spine should be followed and examined for regression of the cystic lesions and improvement of enhancement. Nerve clumping or displacement of the nerve roots may persist presumably because of adhesive arachnoiditis and scarring.^29^ CSF analysis can reveal cellularity and hypoglycorrhachia, but with successful medical therapy, it should normalize. Endpoints for therapy have not been established, but as in SAN of the brain, close follow-up of the patient while tapering corticosteroids are important. Surgical treatment is indicated in the management of acute symptomatic disease to relieve mass effect, as well as in patients who have mass effect and experience severe and progressive neurological dysfunction despite corticosteroids. Cysts degenerating in the spine can elicit inflammation and scarring, making excision of these lesions technically difficult. Subarachnoid scarring may be severe enough to obstruct CSF flow necessitating duraplasty to reestablish CSF flow. Treatment regimens are not standardized; therefore, close follow-up of the patient is critical as corticosteroids are tapered. Surgical treatment with excision of the cysticerci after laminectomy has been the norm in cases of spinal cord or radicular compression. Surgical removal in the setting of severe arachnoiditis, where cysts have adhered to the sacral roots and adjacent dura can be very difficult and in some cases, complete removal of the cysts is impossible. Unless the surgeon is confident that the cysticerci have been completely removed, patients should be treated with antiparasitic drugs postoperatively and undergo follow-up with imaging and CSF analysis as outlined previously for intracranial subarachnoid cysticercosis.

## RECOMMENDATIONS FOR MANAGEMENT OF OCULAR CYSTICERCOSIS (OC)

### XXV. What is the optimal management of OC?

#### Recommendation.

46. We suggest that intraocular cysticerci should be treated with surgical removal rather than with antiparasitic drugs (weak, low).

#### Evidence summary.

Eye involvement in cysticercosis may include the extraocular muscles or subconjunctiva. Intraocular disease may involve the anterior chamber, vitreous, or subretinal location. Orbital, extraocular muscle, and subconjunctival cysticercosis are often amenable to medical therapy. Intraocular cysticercosis has traditionally been approached surgically.^145,146^ Untreated intraocular disease was thought to be a contraindication to cysticidal therapy due to the risk of inducing inflammation, which can lead to blindness. Although there are anecdotal reports of successful medical therapy of ocular NCC,^147,148^ there are not sufficient data on the safety of this approach. Thus, at the present time, intraocular cysticerci should be treated by surgical removal.

## RECOMMENDATIONS FOR THE TREATMENT OF SPECIAL POPULATIONS (SP)

### XXVI. Should children be managed differently from adults?

#### Recommendation.

47. There is no evidence that management of NCC in children should be different from adults with the same form of disease (strong, moderate). Dosing should be weight based.

#### Evidence summary.

The clinical spectrum of NCC is somewhat different in children from adults.^54,149^ Children are more likely to present with SEL and cysticercal encephalitis. Both albendazole and praziquantel have been used safely in millions of children aged more than 1 year. The pharmacokinetics of the drugs is similar to those seen in adults. Because of the incubation period between infection and onset of symptoms, it is rare for NCC to present in infants and there are limited data on drug safety, pharmacokinetics, or efficacy in this population.

### XXVII. Should management be different in pregnant women?

#### Recommendation.

48. We suggest that antihelminthic therapy should be deferred until after pregnancy (weak, low). *Remarks*: Pregnant patients with elevated intracranial pressure need to be aggressively managed as they would be, if not pregnant. Corticosteroids can be used in pregnancy when necessary. The use of antiepileptic drugs should take into account altered pharmacokinetics and potential teratogenicity. Phenobarbital and valproic acid are known to have high rates of teratogenicity. Antihelminthic drugs are rarely required emergently and their use can usually be deferred until after delivery. Methotrexate is teratogenic and should be avoided.

#### Evidence summary.

The management of NCC in pregnant women presents challenges because of concerns about teratogenicity of the drugs. Patients who are symptomatic and have significant morbidity should receive optimal symptomatic therapy. Pregnant patients with elevated intracranial pressure need to be aggressively managed as they would be, if not pregnant. The use of antiepileptic drugs should take into account altered pharmacokinetics and potential teratogenicity. Valproate is associated with a higher rate of teratogenicity. Otherwise, the choice of antiepileptic drugs should follow the guidelines for the management of epilepsy in pregnancy.

By contrast, antihelminthic drugs are rarely required emergently, and their use can usually be deferred until after delivery. Praziquantel is a category B agent with no clear associated teratogenicity in animal studies and limited data suggesting that it is safe in pregnancy.^150^ Based on teratogenicity observed in animal models, albendazole is not considered safe in pregnancy.^151^ However, limited data are emerging that treatment of pregnant women with albendazole for intestinal helminths actually improves birth outcomes without evidence of increased rates of birth defects.^152^ In general, cysticidal therapy should be deferred until after delivery.

Corticosteroids can be used in pregnancy when necessary. Methotrexate is teratogenic and should be avoided.

## Supplementary Material

Supplemental Tables.
