# Supplementary material for: Diagnosis and Treatment of Neurocysticercosis: 2017 Clinical Practice Guidelines by the Infectious Diseases Society of America (IDSA) and the American Society of Tropical Medicine and Hygiene (ASTMH)
Source: Am J Trop Med Hyg. 2018 Apr;98(4):945–66. doi: 10.4269/ajtmh.18-88751 (PMC5928844; doi:10.4269/ajtmh.18-88751)
Supplement: Supplementary file 1 [file tpmd1888751.SD1.pdf]

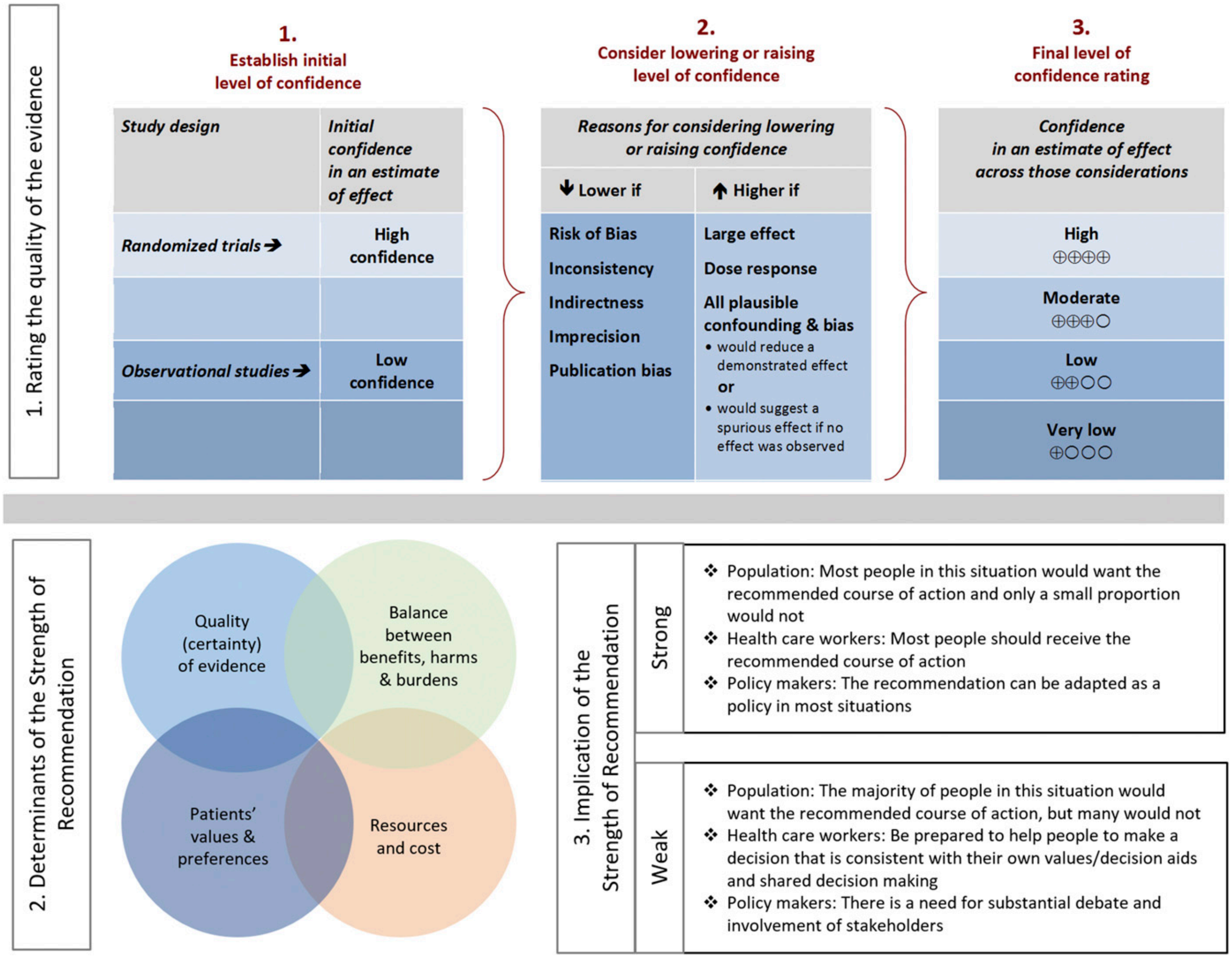

FIGURE 1. Approach and implications to rating the quality of evidence and strength of recommendations using the Grading of Recommendations, Assessment, Development and Evaluation (GRADE) methodology (unrestricted use of the figure granted by the U.S. GRADE Network).

Table 1

Classification of NCC based on location and appearance of the parasite and surrounding host tissue on neuroimaging

Form*	Characteristic on neuroimaging	Histopathology
Parenchymal†		
Nonviable calcified NCC (CPN)	Nodular calcifications less than 20 mm in diameter (often 1–5 mm) with or without surrounding edema and/or contrast enhancement.	Calcified granuloma with or without surrounding inflammation and/or gliosis.
Single, small enhancing (single enhancing lesions, SEL)	Cystic or nodular enhancing lesion < 2 cm in size.	Single parenchymal parasites in the process of degeneration with surrounding inflammation and variable opacification or absence of the cyst fluid.
Viable parenchymal NCC	Vesicular lesions often with evidence of associated contrast enhancement and/or surrounding edema. The scolex is often visible on high-definition imaging.	Parasites with intact cyst wall, vesicular fluid, and scolex, with variable amounts of inflammation surrounding the parasite sometimes invading the cyst wall.
Extra-parenchymal†		
Intraventricular NCC	Cysticerci within the ventricles, obstructive hydrocephalus or loculated hydrocephalus with disproportionate dilatation of the ventricles in computed tomography/magnetic resonance imaging suggestive of a cysticercus).	Viable cysticercus cyst within the ventricle and/or obstructive hydrocephalus.
Subarachnoid NCC	Cysticerci in the Sylvian fissure, in the basilar cisterns, or inter-hemispheric spaces. Strokes or meningitis without discrete cysts.	Cysticerci in the subarachnoid space often with arachnoiditis, vasculitis. The cysticerci are often in clusters with proliferating membranes (racemose) and may lack a scolex.
Spinal NCC	Cysticerci within the spinal subarachnoid space with or without evidence of inflammation/diffuse spinal arachnoiditis. Intramedullary cysticerci within the spinal cord.	Subarachnoid cysticerci often with associated arachnoiditis. Intramedullary cysticerci similar pathologically to parenchymal cysticerci.

NCC = neurocysticercosis.

* Patients with more than one form are classified with the form found lower on the chart with the exception that SEL that are also viable are grouped with SEL. Ocular cysticercosis is classified separately.

† Refers to cysticerci in the brain parenchyma. Small cysticerci in the gyri over the cerebral convexity behave clinically like parenchymal cysticerci and are grouped with parenchymal cysticerci. Rare forms of NCC include multiple inflamed parenchymal cysticerci with diffuse cerebral edema, termed cysticercal encephalitis, large parenchymal cysticerci (> 20 mm).

TABLE 2
Summary of treatment recommendations for different forms of parenchymal NCC

Form	Type of therapy/subgroup	Recommendation	Comment	Strength of recommendation; quality of evidence
Viable parenchymal NCC (VPN)	Antiparasitic therapy	Antiparasitic drugs should be used in all patients with VPN unless there is increased intracranial pressure	The preponderance of studies demonstrated more rapid radiologic resolution in patients treated with antiparasitic drugs compared with placebo and decreased numbers of generalized seizures*	Strong; moderate
	1–2 viable cysts	Monotherapy with albendazole (15 mg/kg/d in two daily doses up to 1,200 mg/day) with food for 10 days	Combination therapy showed no additional benefit with one or two cysts and more complex pharmacology†	Strong; moderate
	> 2 viable cysts	Albendazole (15 mg/kg/day in two daily doses up to 1,200 mg/day) combined with praziquantel (50 mg/kg/d in three daily doses) for 10 days	Both the pharmacokinetic study and a recent randomized trial demonstrated improved radiologic resolution with the combination compared with albendazole alone in those with more than two cysticerci†	Strong; moderate
	Anti-inflammatory therapy	Corticosteroids should be used whenever antiparasitic drugs are used	Adjuvant use of corticosteroids is associated with fewer seizures during therapy. Optimal doses have not been clearly defined‡	Strong; moderate
	Antiepileptic therapy	Antiepileptic drugs should be used in all patients with seizures	Antiepileptic drugs appear to be effective in controlling seizures in patients with parenchymal NCC; consider tapering off after 2 years if meet criteria for withdrawal as in idiopathic epilepsy§	Strong; moderate
Single enhancing lesion (SEL) due to NCC	Antiparasitic therapy	Albendazole (15 mg/kg/day in two daily doses up to 800 mg/day) for 1–2 weeks	Albendazole shown to improve seizure outcome in meta-analyses.‖ Different studies have used a range of durations of treatment without clear advantages of longer duration.	Weak; high
	Anti-inflammatory therapy	Corticosteroids should be given concomitantly with antiparasitic agents‖	Given the data on worsening symptoms with antiparasitic drugs, most authorities recommend the use of corticosteroids in patients treated with antiparasitic drugs¶	Strong; moderate
	Antiepileptic therapy	Antiepileptic drugs (AEDs) should be used in all patients with seizures#	Antiepileptic drugs can be discontinued after resolution of cystic lesions if no risk factors for recurrence.# Risk factors for recurrent seizures include: 1) calcifications on follow-up computed tomography (CT), 2) breakthrough seizures, and 3) > 2 seizures during the course of the disease.	Strong; moderate
Calcified parenchymal NCC with or without perilesional edema	Antiparasitic therapy	Antiparasitic treatment not recommended	There are no viable cysts and thus no indication for antiparasitic therapy	–
	Antiepileptic therapy	Treatment with AEDs#	Management guidelines are similar to that in other patients with seizures	Strong; moderate
	Anti-inflammatory therapy	Corticosteroids should not be routinely used**	There are a few case reports suggesting that when corticosteroids are stopped or lowered previously quiescent calcifications develop perilesional edema	Strong; low

(continued)

Table 2
Continued

Form	Type of therapy/subgroup	Recommendation	Comment	Strength of recommendation; quality of evidence
Cysticercal encephalitis (with diffuse cerebral edema)	–	Avoid antiparasitic drugs and treat diffuse cerebral edema with corticosteroids††	Cerebral edema mediated by the host inflammatory response. Antiparasitic drugs are associated with worsening edema	Strong; low

NCC = neurocysticercosis.

* Two well-designed randomized trials demonstrated more rapid radiologic responses and fewer generalized seizures in patients treated with albendazole compared with placebo.

† The combination of praziquantel and albendazole was superior to albdendazole alone in patients with more than two cysts, but was not better in those with one or two viable cysts.

‡ The optimal anti-inflammatory regimen has not been clearly defined. A trial comparing 6 mg/day of dexamethasone for 10 days with 8 mg/day for 28 days followed by a taper noted fewer seizures in the higher dose group. Other studies have used prednisone 1–1.5 mg/kg/day during therapy.

§ There are no clear data on optimal duration of antiepileptic drugs. Risk factors for recurrent seizures include calcifications on follow-up CT, breakthrough seizures, and > 2 seizures during the course of the disease. In patients without any of these risk factors and no seizures in the prior 3 months, AEDs can be safely withdrawn within a few weeks of the resolution of the SCG on high resolution imaging studies.

‖ Two meta-analyses of randomized controlled trials have concluded that albendazole improved the outcome in patients with SEL due to NCC.

¶ Albendazole should be given along with anti-inflammatory drugs. The optimal dose and duration has not been defined, but doses have included dexamethasone 0.1 mg/kg/day for the duration of therapy or 1–2 mg/kg/day of prednisone or prednisolone have been used.

# Management guidelines are similar to that in other patients with seizures. Many can be managed with a single drug. There are no data on relative efficacy of different antiepileptic drugs.

** A few case reports suggest when corticosteroids are lowered or stopped, rebound perilesional edema can occur. Therefore, anti-inflammatory drugs should be used cautiously, if at all, in patients presenting with perilesional edema around a calcified lesion.

†† Antiparasitic agents can worsen cerebral edema and should generally be avoided in patients with increased intracranial pressure from either diffuse cerebral edema (cysticercal encephalitis) or untreated hydrocephalus.

Table 3
Recommendations for therapy of extra-parenchymal NCC

Form	Recommended therapy	Comment	Strength recommendation; quality of evidence
Intraventricular NCC (IVN, lateral or third ventricle)	Removal of the cysticerci by minimally invasive, neuroendoscopy when feasible*†	Most cases with isolated nonadherent cysts in the lateral or third ventricle can be cured by neuroendoscopy and do not require subsequent antiparasitic drugs or shunt therapy if all cysticerci removed.	Strong; low
IVN (fourth ventricle)	Either endoscopic or microsurgical cystectomy is suitable, depending on the experience of the surgeon	Microsurgical resection is from a suboccipital approach. The endoscopic approach can be either from the conventional lateral-third ventricular-trans-aqueductal route (technically demanding) or through the posterior approach.	Strong; low
IVN—when surgical removal not feasible (e.g., adherent cyst)	Cerebrospinal fluid (CSF) diversion via a ventriculoperitoneal shunt‡	In cases of marked inflammation in the ventricles or degenerating cysticerci, the cyst may adhere to the ventricular wall, making removal hazardous. Cerebrospinal fluid diversion with medical therapy is the recommended approach.‡	Weak; low
	Adjuvant antiparasitic and anti-inflammatory therapy‡	Medical therapy should be limited to patients in whom surgery is contraindicated because of various reasons. A CSF diversion (shunt) should always be performed before chemotherapy if there is hydrocephalus because there are reports of precipitation of hydrocephalus with antiparasitic therapy.	Strong; moderate
Subarachnoid NCC§ (SAN)	Surgical management of hydrocephalus	Initial management should focus on treatment of hydrocephalus. This often requires ventriculoperitoneal shunting.	Strong; low
	Antiparasitic therapy‖	Subarachnoid cysts do not respond well to typical doses and durations of therapy. Options to improve responses include prolonged administration of albendazole (15 mg/kg/day for months) or combination therapy with albendazole (15 mg/kg/day plus praziquantel 50 mg/kg/day.	Strong; low
	Anti-inflammatory therapy	Concomitant administration of corticosteroids with antiparasitic drugs is essential in the treatment of patients with SAN. Inflammation is exacerbated as a result of antiparasitic treatment.¶	Strong; low

NCC = neurocysticercosis.
* The endoscopic surgical approach often requires ventriculomegaly. Cyst rupture is the norm and not associated with adverse consequences. The microsurgical approach is also facilitated by the presence of hydrocephalus.
† Alternative approaches include 1) CSF diversion along with medical management or 2) craniotomy with microsurgical excision.
‡ Shunts are initially efficacious acutely for hydrocephalus, but there is a very high rate of shunt malfunction in patients with NCC. Shunt failure may be lower when combined with corticosteroids and antiparasitic treatment.
§ SAN should be aggressively treated with antiparasitic and anti-inflammatory drugs. Hydrocephalus should be addressed before antiparasitic therapy.
‖ Untreated hydrocephalus is a contraindication to antiparasitic therapy and needs to be treated first. Some cases respond to anti-inflammatory treatment, but most cases require CSF diversion.
¶ Generally, 1 mg/kg/day of prednisone or 0.2–0.4 mg/kg/day of dexamethasone are administered 3–4 days before and during antiparasitic treatment. The dose is slowly decreased, depending on the intensity of the inflammatory response. Methotrexate or antibody to TNF can be used as steroid-sparing agents.

Table 4

Clinical pearls for management of neurocysticercosis (NCC)

NCC is a spectrum of diseases that differ in pathogenesis and optimal management.
Symptomatic therapy* should be the focus of initial and emergency management.
Antiparasitic treatment is important, but never an emergency.
Parenchymal cystic NCC has better outcomes if treated with antiparasitic drugs along with corticosteroids.
Subarachnoid NCC (SAN) does not respond well to single antiparasitic drugs at doses and durations used for parenchymal NCC. Optimal management may require chronic anti-inflammatory therapy, intensive antiparasitic therapy†, and surgical therapy.‡
Ventricular NCC of third and lateral ventricles should be treated with minimally invasive surgery when possible,§ but minimally invasive and open craniotomy are options for fourth ventricular disease. Open craniotomy or cerebrospinal fluid (CSF) diversion along with antiparasitic drugs are optimal in select cases. Antiparasitic therapy should be deferred until after surgical therapy.
Calcified lesions do not contain viable parasites and should not be treated with antiparasitic drugs.

* Symptomatic therapy includes antiepileptic drugs for seizures, anti-inflammatory drug such as corticosteroids and methotrexate, and surgery for hydrocephalus.

† Antiparasitic therapy for SAN may include prolonged courses of albendazole, high-dose albendazole, or combinations of praziquantel and albendazole.

‡ Surgical therapy for SAN may include CSF diversion for hydrocephalus or minimally invasive surgical debulking.

§ Adherent cysticerci should be managed with CSF diversion along with antiparasitic drugs. Open craniotomy is effective for fourth ventricular lesions and the choice of approaches should depend on local surgical expertise.
